# Seeing the forest for the scenarios: Using decision trees to draw conclusions from large energy scenario sets

**DOI:** 10.1016/j.isci.2026.117079

**Published:** 2026-09-30

**Authors:** Florian Baader, Stefano Moret, Wolfram Wiesemann, Iain Staffell, André Bardow

**Affiliations:** 1 Energy and Process Systems Engineering, Department of Mechanical and Process Engineering, ETH Zürich, Zürich, Switzerland; 2 Imperial College Business School, Imperial College London, London, UK; 3 Centre for Environmental Policy, Imperial College London, London, UK; 4 Department of Management, Economics and Industrial Engineering, Politecnico di Milano, Milan, Italy

**Keywords:** energy planning, energy systems, modeling, machine learning, decision-making, decision trees, uncertainty, stakeholders

## Abstract

Energy transition studies often present hundreds of scenarios, yielding large datasets that are challenging to interpret. Alternative approaches define a few qualitative storylines, which are more accessible but disconnected from model-based evidence. Here, we show that decision trees, a popular machine learning technique, bridge this disconnect by distilling numerous scenarios into a small set of interpretable storylines centered around policy-relevant levers. We demonstrate this approach across three global and European case studies: First, we translate 1000 integrated assessment model scenarios into three storylines distinguished by their degrees of heating and industry electrification. Second, we show that 2°C warming pathways with continued fossil fuel usage only work under unusually mild climate responses. Third, we use 441 European pathways to show that high bioenergy or electrification are required for cost-effective climate neutrality. Our transferable approach helps decision-makers interpret large scenario sets by revealing how policy-relevant outputs are associated with different transition pathways.

## Highlights

• Large numbers of “what-if” energy scenarios can overwhelm decision-makers
• Decision trees translate many energy scenarios into a few interpretable storylines
• The method helps stakeholders navigate complex trade-offs in the energy transition
• Three case studies demonstrate the approach at European and global scale

## Graphical abstract

**Figure:**
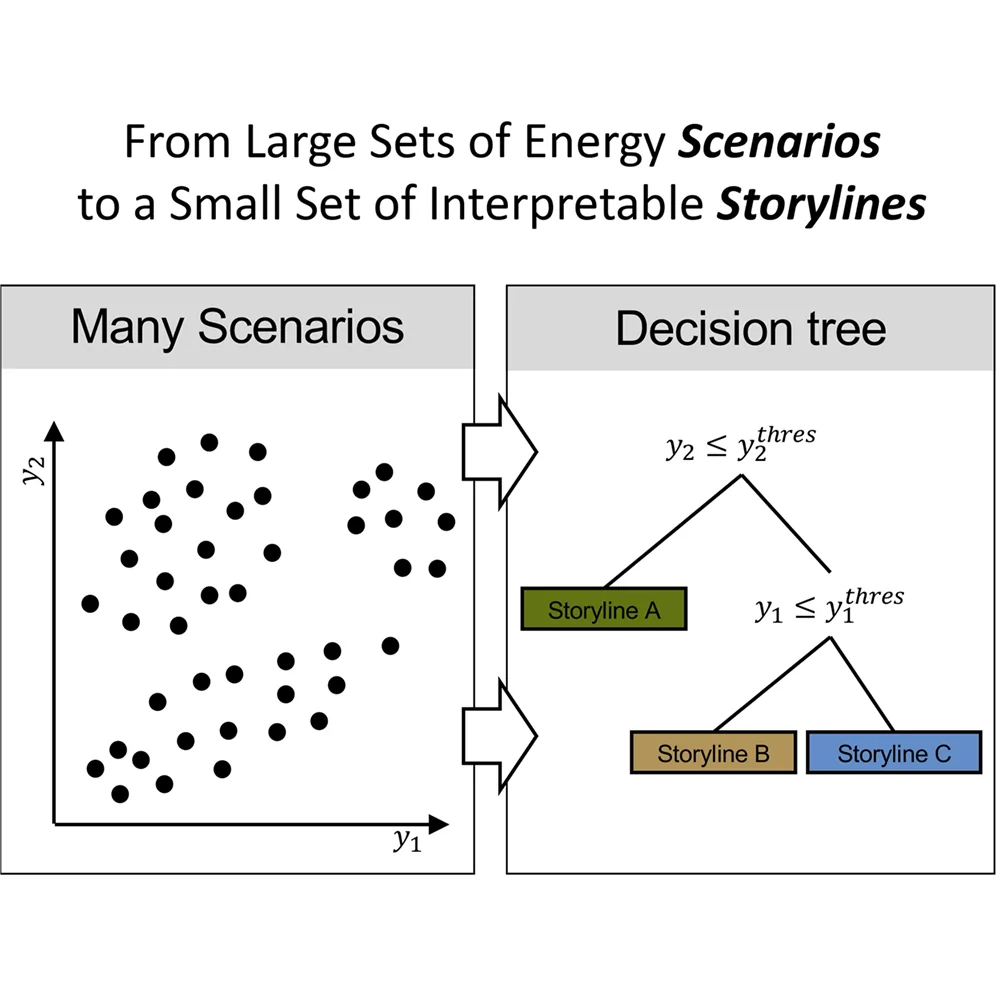

## Teaser

Energy sustainability; Energy Resources; Energy systems; Energy management

## Introduction

As global surface temperatures have reached 1.5°C above pre-industrial levels,^1^ all sectors must urgently pursue deep and sustained greenhouse gas emission reductions to secure a liveable and sustainable future for humanity.^2^

However, despite the stark reality of climate change, the availability of mature technological solutions and clear evidence that immediate action is more beneficial than a wait-and-see strategy,^3^^,^^4^^,^^5^^,^^6^ the Intergovernmental Panel on Climate Change’s (IPCC) latest report warns that “the pace and scale of what has been done so far, and current plans (to tackle climate change), are insufficient”.^7^

This indecisiveness in policy deployment,^8^ resulting in the loss of vital time, can in part be attributed to the substantial uncertainty and the complex trade-offs affecting long-term energy systems planning and decision-making. Such trade-offs include the need to balance potentially conflicting objectives such as energy security, equity, and sustainability in the energy transition.^9^

Models can help decision-makers explore these trade-offs, for example, by assessing the impact of the energy transition on relevant societal (e.g., creation of jobs^10^^,^^11^ and social justice^12^) or environmental concerns (e.g., biodiversity loss^13^). However, accounting for these additional dimensions and considering uncertainties often leads to complex studies that overwhelm decision-makers with vast numbers of computer-generated scenarios. This practice is in contrast with the needs of stakeholders and decision-makers, who look for a manageable number of options to evaluate^14^ and whose preferences may not be captured by quantitative models. This gap between quantitative models and real-world decision-making processes exacerbates the difficulty of creating consensus around science-based energy and climate mitigation policies.^15^

In this paper, we narrow this gap by introducing a method that streamlines hundreds or thousands of energy scenarios into a few interpretable storylines. Specifically, we highlight the potential of training machine learning algorithms on key model outputs of interest that correspond to policy-relevant levers, which allows us to show how these levers are interconnected in the energy transition. The outputs of interest can be chosen by decision-makers to answer specific questions. We first apply decision trees to a subset of the AR6 scenario database, a comprehensive ensemble of quantitative, model-based pathways underpinning the Sixth Assessment Report (AR6) of Working Group III by the IPCC,^2^^,^^16^ where we additionally demonstrate that the method can systematically identify model fingerprints in large model ensembles, as recently found by Dekker et al.^17^ Then, applications to recently published results for both the global energy transition (using an Integrated Assessment Model under uncertainty^18^) and to the European energy transition^19^ demonstrate that the wide solution space can be reduced to a small number of storylines depending on the values of specific outputs. Additionally, we propose a new way to visualize these choices as decision trees, illustrating how the scenario space progressively shrinks by conditioning on policy-relevant outputs of interest. This representation highlights the most important interconnections and trade-offs among key drivers of the energy transition.

Overall, decision trees can help bridge the disconnect between model-based and stakeholder-driven decision processes in the energy transition, informing stakeholders with interpretable storylines backed up by quantitative and transparent models.

### Complexity as a barrier between models and decision support

*Quantitative* models have been developed since the 1970s to assist decision-makers in the definition of climate change mitigation pathways.^20^^,^^21^^,^^22^ For example, integrated assessment models (IAMs) represent the complex interactions and long-term feedbacks between the socioeconomic system and the natural system to inform climate policy-making^23^; Energy models are widely used to devise investment and operation strategies for energy and power systems.^24^^,^^25^^,^^26^^,^^27^

Given the large uncertainties associated with long-term energy planning,^28^ in recent years, these models have been extended to factor in parametric uncertainty^29^^,^^30^^,^^31^ and to systematically explore different options, e.g., by adopting modeling to generate alternatives (MGA) methods.^19^^,^^32^^,^^33^^,^^34^^,^^35^ While informative and thorough, uncertainty and MGA methods, which have also been used in combination,^36^^,^^37^^,^^38^ often result in hundreds of scenarios and complex trade-offs that are difficult to interpret for decision-makers. For example, Pickering et al.^19^ find 441 technically feasible and cost-effective options to reach carbon neutrality in Europe, while Neumann et al.^39^ generate 1968 near-optimal alternatives for a completely renewable European electricity system. Similarly, the outcome of uncertainty studies may not be easily accessible: uncertainty and sensitivity analyses^29^^,^^40^ run models hundreds of times to calculate probability distributions for the main outputs of interest.

Another strategy to address uncertainty in complex systems is using model ensembles, which aim to reduce model uncertainty and increase robustness by running several models for a common set of experiments.^41^^,^^42^ This approach has found wide application in climate science and constitutes the basis of the AR6 database underpinning the Sixth Assessment Report (AR6) of Working Group III by the IPCC.^2^^,^^16^ However, model ensembles are similarly affected by difficulties in comparison and interpretation of the spread of their outcomes,^43^ which has generated a new stream of literature aiming to unpack meaning from ensemble studies, e.g., by using statistical learning^44^ or decomposition methods.^45^^,^^46^

In summary, especially when extended to include parametric and model uncertainty, energy transition studies often generate hundreds of plausible scenarios. While several existing approaches help extract insights from these large scenario sets—for example by identifying must-haves, must-avoids, ranges, correlations, or clusters—we provide a useful complement by streamlining this information to make it more accessible to decision-makers.

An alternative established approach to guide energy transition decisions relies on socio-economic “storylines”, which are *qualitative* descriptions of energy system configurations,^47^ typically outlined in a participatory process with relevant stakeholders.^48^ After defining the storylines, calculations are performed using a quantitative model, following the “story-and-simulation” method.^49^^,^^50^^,^^51^

This approach comes with important benefits: storylines can cover broader contextual developments that may not be part of the models, and importantly, the qualitative nature of storylines makes them accessible and interpretable to a broader public. Additionally, engagement of stakeholders allows accounting for “soft” aspects, which are difficult to capture in models, and can foster trust and ownership in the process.^52^ An example of active involvement of stakeholders is the process adopted by the UK National Energy System Operator for the definition of their Future Energy Scenarios.^53^ And, especially in larger research consortia, focusing on a small number of representative storylines helps to align research. The largest example is likely the Shared Socioeconomic Pathways (SSPs)^54^ adopted by the IPCC, where five narratives have been translated into a scenario framework to serve as a common basis for scientific debate.^55^^,^^56^^,^^57^

Defining these common assumptions and storylines is often a qualitative process requiring multiple iterations. Also, despite their several advantages, storylines present the risk of being biased toward the interests of dominant stakeholders,^48^ and a fundamental challenge is represented by their internal consistency and representativeness.^58^^,^^59^ In addition, qualitative storylines received from stakeholder interviews may prove infeasible when quantified with energy models.^60^

Effectively coupling the quantitative power of energy models with the broader accessibility of storylines has become of increasing interest in recent years.

In this context, the story-and-simulation literature has evolved toward iterative approaches to reconcile the possible discrepancies between socioeconomic storylines and model outcomes.^60^^,^^61^^,^^62^ However, these approaches still require initial qualitative inputs as well as the definition of iteration procedures, and hence do not fully get away from the risks of biases and incompleteness associated with the socio-economic approach. Participatory modeling^63^^,^^64^ and human-in-the-loop^35^ approaches have been proposed to better integrate stakeholder needs in MGA studies, but still result in many scenarios which need to be streamlined for interpretability.

Another research trend exploits the exploratory nature of energy models by generating a large number of model runs that are then aggregated into a small number of similar clusters.^65^ Clustering can also be used in combination with “scenario discovery”,^66^^,^^67^^,^^68^ which uses simulation models as scenario generators to identify the combination of uncertainties under which particular model behavior occurs.^69^^,^^70^^,^^71^ While linking uncertain inputs to clusters of scenarios helps to explore the solution space and understand the effect of uncertain drivers, such clustering offers limited insight for decision-making because the realization of these uncertainties cannot be anticipated.

### Decision trees translate many scenarios into storylines

To narrow this gap between quantitative and qualitative approaches to inform decision making, we introduce a decision tree method that disentangles large numbers of scenarios into a handful of storylines. The steps of the methods are outlined conceptually in Figure 1, while a detailed description is available in the STAR Methods section. The method starts from the definition of the outputs of interest **y**; importantly, the selected output of interest should align with policy-relevant levers. For example, if decision-makers can influence the penetration of electric mobility (e.g., through subsidies or regulation), the share of electric mobility is a meaningful output. Then, several scenarios, often a few hundred, are generated by running models, with each scenario representing a possible future of the system. Note that, as we do in this paper, such scenarios may also be drawn from existing databases or literature sources. The scenarios are then grouped using clustering techniques, and finally, a decision tree, with the number of leaves fixed to match the number of clusters, is trained to predict the cluster based on the outputs of interest **y**. The final result is a tree that organizes the scenario space: the nodes represent thresholds on the outputs of interest used to partition the scenario ensemble, while the leaves are the clusters of scenarios that define distinct energy transition storylines.

**Figure 1:**
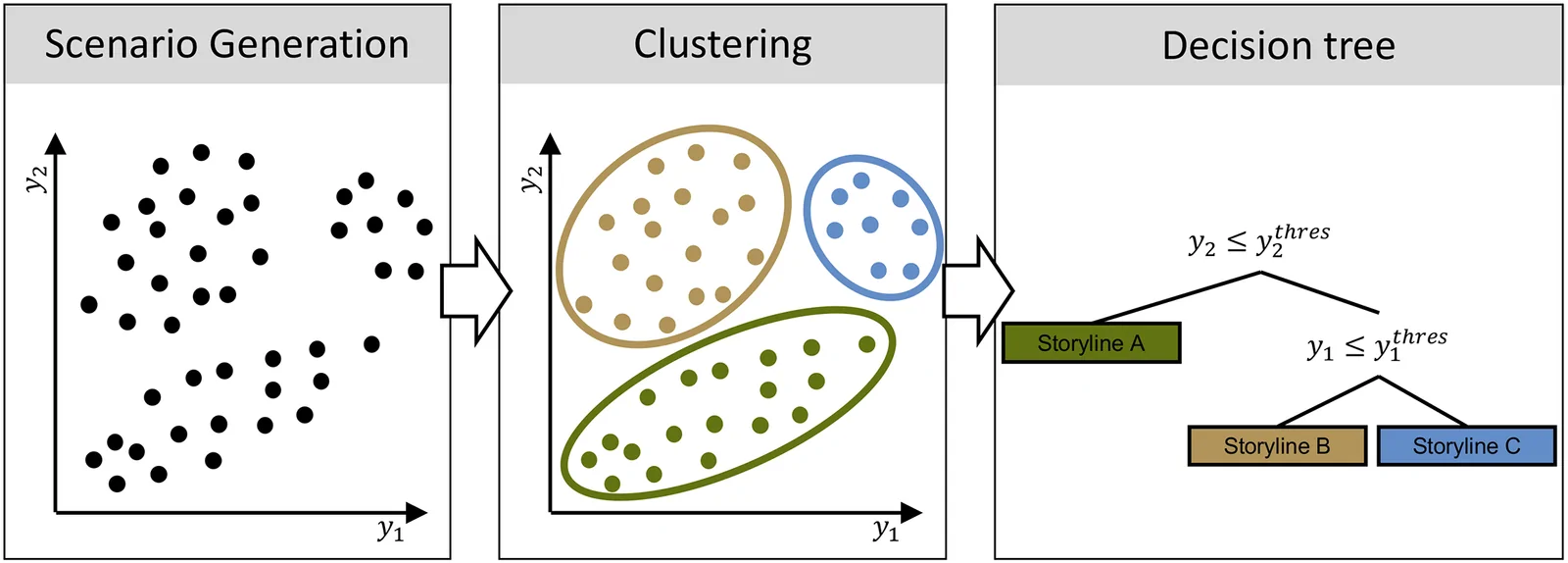
Conceptual outline of the proposed decision tree method to streamline large numbers of scenarios into storylines, using only two outputs of interest: *y*_1_ and *y*_2_ After the definition of the outputs of interest **y**, scenarios are generated using quantitative models. Then, clustering splits the data points into three clusters, corresponding to energy transition storylines. Finally, a decision tree is trained to predict the storyline based on the outputs of interest **y**.

## Results

### *Global model ensembles*

We apply our method to the global energy transition, first on a subset of the AR6 scenario database informing the corresponding IPCC report,^16^ and then on a recently published study of global decarbonization pathways under uncertainty.^18^

The AR6 database consists of approximately 1200 scenarios generated by different IAMs and categorized by their climate targets. In the following, we demonstrate the method on the C2 scenarios (i.e., returning to 1.5°C in 2100 after a high overshoot) that are also part of the ENGAGE model intercomparison study.^6^ The resulting dataset comprises 56 scenarios generated using six different IAMs, to which we applied the debiasing procedure proposed by Dekker et al.^45^ to remove biases stemming from the uneven representation of models in the ensemble.

We focus on model outcomes that can correspond to different policy directions; specifically, we study the primary energy supply mix and the technology choices for the transition of the industry, heating and transport sectors in 2100. These outputs of interest vary significantly across the scenarios, which makes it difficult to derive clear guidance. In particular, for primary energy, the renewable energy supply varies between 532 and 1131 EJ/y, and the primary fossil energy supply varies from 11 to 420 EJ/y. For sector coupling in the three main non-electricity sectors, we consider the following outputs of interest: electrification of industry (varying between 42% and 83%), electrification in the residential and commercial sectors (60%–92%), and the share of non-fossil transport, i.e., based on electricity, biofuels or hydrogen (37%–92%). A systematic procedure for the choice of the outputs of interest is proposed in the STAR Methods section.

Figure 2 shows the resulting decision tree for this dataset. At the root node lies the full solution space, with the radar plots and the open locks indicating the large ranges of variations in the C2 scenarios. The decision tree clusters these scenarios into four storylines determined by three outputs of interest: (1) share of non-fossil transport, (2) electrification of industry, and (3) penetration of renewables (Figure 2a). Additionally, we find a strong correlation between the four identified storylines and the different models used to generate the AR6 scenarios (Figure 2b), clearly unveiling the “model fingerprints”.^17^

**Figure 2:**
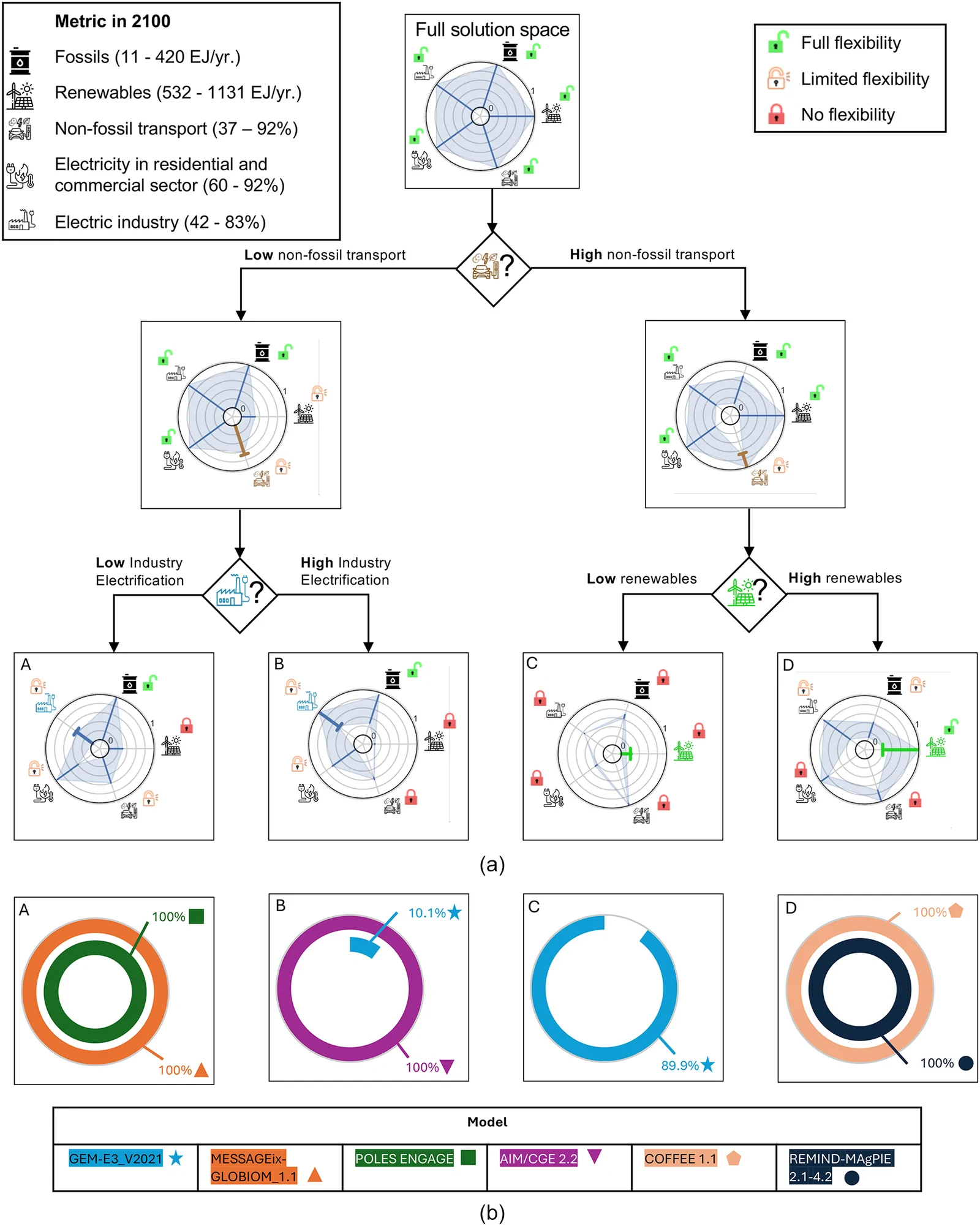
Decision tree for the global model ensemble (a) Decision tree translating the C2 scenarios from the ENGAGE model comparison within the AR6 Scenarios Database into four storylines defined by three policy-relevant levers (share of non-fossil transport, electrification of industry, and penetration of renewables) along five outputs of interest. The axes of the radar plots are normalized on the range of each output of interest, while the locks indicate the room for maneuver available. (b) Correlation between the four identified storylines (A, B, C, D) and the different models used to generate the AR6 scenarios, clearly showing the effect of the “model fingerprints” (e.g., all the scenarios from the COFFEE and the REMIND-MAgPIE models are found in storyline D).

#### Global decarbonization pathways under uncertainty

As a second case study, we apply our method to the global decarbonization pathways under uncertainty studied by Panos et al.,^18^ who recently presented a Monte-Carlo assessment of an integrated assessment model (IAM). Differently from the AR6 database, where several IAMs are run for a common set of experiments in a deterministic setting (i.e., without accounting for parametric uncertainty), Panos et al.^18^ use only one model (ETSAP-TIAM) but account for different sources of uncertainty. Using this IAM, they devise energy transition pathways under uncertainty by defining 18 key uncertain parameters, including energy system, economic, and climate drivers, which are then sampled in a Monte Carlo analysis.

Here, we focus on the 1000 Monte Carlo scenarios calculated with the constraint that the global average temperature in 2100 does not exceed 2°C above the pre-industrial level. For consistency with our previous results, we focus on the same set of model outcomes, with the exception of low-temperature heat electrification, which is only reported for the residential sector in.^18^

By applying decision trees, these 1000 scenarios can be summarized by only three leaves, corresponding to three storylines for the global energy transition. These storylines are defined by two main drivers: (1) electrification of heating and (2) electrification of industry (Figure 3a). The first driver differentiates between scenarios with low and high shares of electric heating, and thereby splits the set of solutions: A low share of electric heating is associated with low non-fossil transport and minimal electrification of industry. Moreover, deployment of renewables is low. On the other side of the tree, with a high share of electric heating, the share of non-fossil transport is also high.

**Figure 3:**
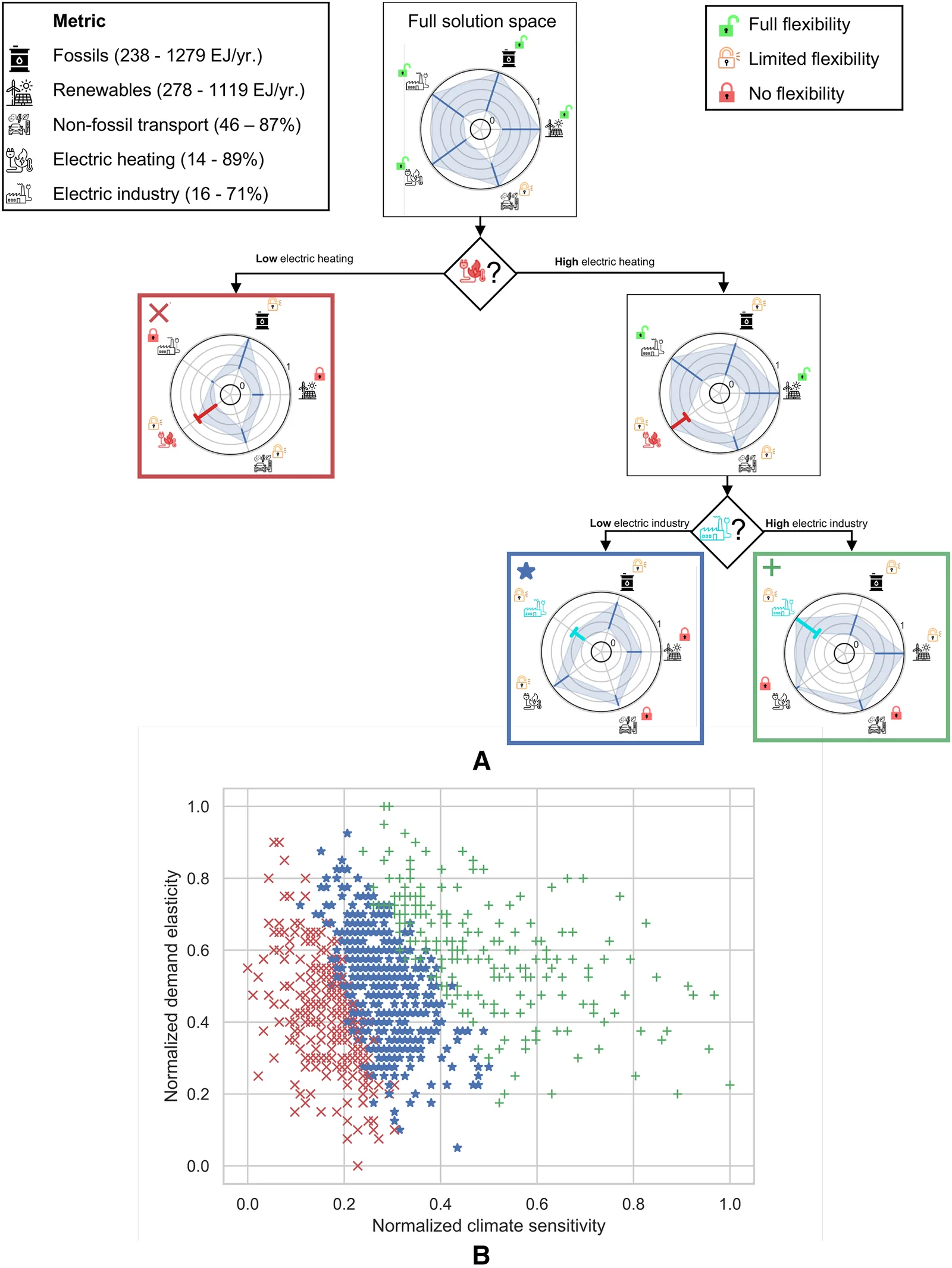
Decision tree for the global case study under uncertainty (A) Decision tree translating 1000 2°C scenarios for the global energy transition in 2100 from the ETSAP-TIAM model^18^ defined by two key outputs of interest (electrification of heating and industry). For an explanation of the symbols, see Figure 2. (B) Clustering of the 1000 scenarios along the two uncertain model inputs with the strongest effect on the resulting storyline. The coloring links the scatterplot to the three storylines of the tree in (A): Scenarios with high penetration of fossils and low sector coupling assume low climate sensitivity and low demand elasticity.

As the next key driver, the tree further differentiates between solutions with low and high electrification of industry. High electrification of industry is associated with a high deployment of renewables and maximum electrification of heating. Overall, the extent to which the resulting storylines rely on renewable energy and sector coupling increases from left to right in Figure 3A.

The three storylines identified by the leaves of the decision tree show substantially different global energy systems and, thus, substantially different directions of global strategic actions. However, given the large uncertainties, it is important to understand which uncertain factors make these actions optimal, which can be assessed for this case study as we have access to the underlying assumptions for the uncertain parameters. Employing a scenario discovery approach,^67^ we show that 2 of the 18 uncertain factors considered by Panos et al.^18^ are sufficient to explain how the different Monte-Carlo scenarios are assigned to one of the three storylines (Figure 3B): (1) climate sensitivity, expressing how strongly the global temperature reacts to greenhouse gases (i.e., for the same emissions, a higher climate sensitivity leads to a higher temperature), and (2) elasticity of energy service demands to their drivers, describing how strongly energy demands scale with respect to drivers such as residential floor area, industrial production index, sectoral value-added, GDP, and population. Figure 3B reveals that storylines with high penetration of fossils and low sector coupling assume low climate sensitivity and low demand elasticity. With higher climate sensitivities and demand elasticities, we move toward renewables and sector coupling.

Overall, the tree breaks down the 1000 Monte Carlo scenarios, translating the quantitative output of the IAM study into three storylines corresponding to three different global futures. Moreover, it provides the insight that betting on fossils and low sector coupling implies gambling the 2°C target on the assumption that climate sensitivity and demand increase will be low.

#### The European energy transition

In the third case study, we consider the 441 near-optimal alternatives proposed by Pickering et al.^19^ for the European sector-coupled energy system. All these alternatives consider an energy-self-sufficient and fossil-free Europe; thus, apart from a small fraction of nuclear power, energy is, by design, provided exclusively by renewable energy sources within Europe. Unlike the uncertainty-driven scenario generation in the previous case study, Pickering et al.^19^ use modeling to generate alternatives (MGA) to identify near-optimal solutions, meaning that all alternatives can be chosen without making implicit bets on the realization of the underlying uncertainties. Still, the near-optimal alternatives show significant maneuvering space and present an overwhelming set of 441 solutions to decision-makers and the general public.

Thus, for effective communication, it is essential to streamline the solution space. As in the previous case study, we observe large variations in the outputs of interest: (1) the share of heat electrification varies between 4% and 100%, (2) the share of transport electrification between 53% and 100%, (3) exploitation of bioenergy resources varies from 0% to 100%, (4) average electricity imports per country between 4 and 69 TWh/y, and (5) installed storage capacity between 0.03 and 11 GW. As in the global case studies, the outputs of interest address sector coupling (for the heating and transport sectors) and energy supply (bioenergy use). Moreover, the reliance on electricity imports relates to intensely discussed strategic directions as they show to what extent European countries produce electricity domestically or rely on an integrated electricity market.

The decision tree in Figure 4 shows that the 441 scenarios can be summarized by splitting across three levers: (1) bioenergy use, (2) storage, and (3) heat electrification. The most critical split is on bioenergy use: Even though the entire solution space suggests freedom in all the considered outputs of interest, using a low amount of the available bioenergy resources is only possible under close-to-full electrification in both the heat and transport sectors.

**Figure 4:**
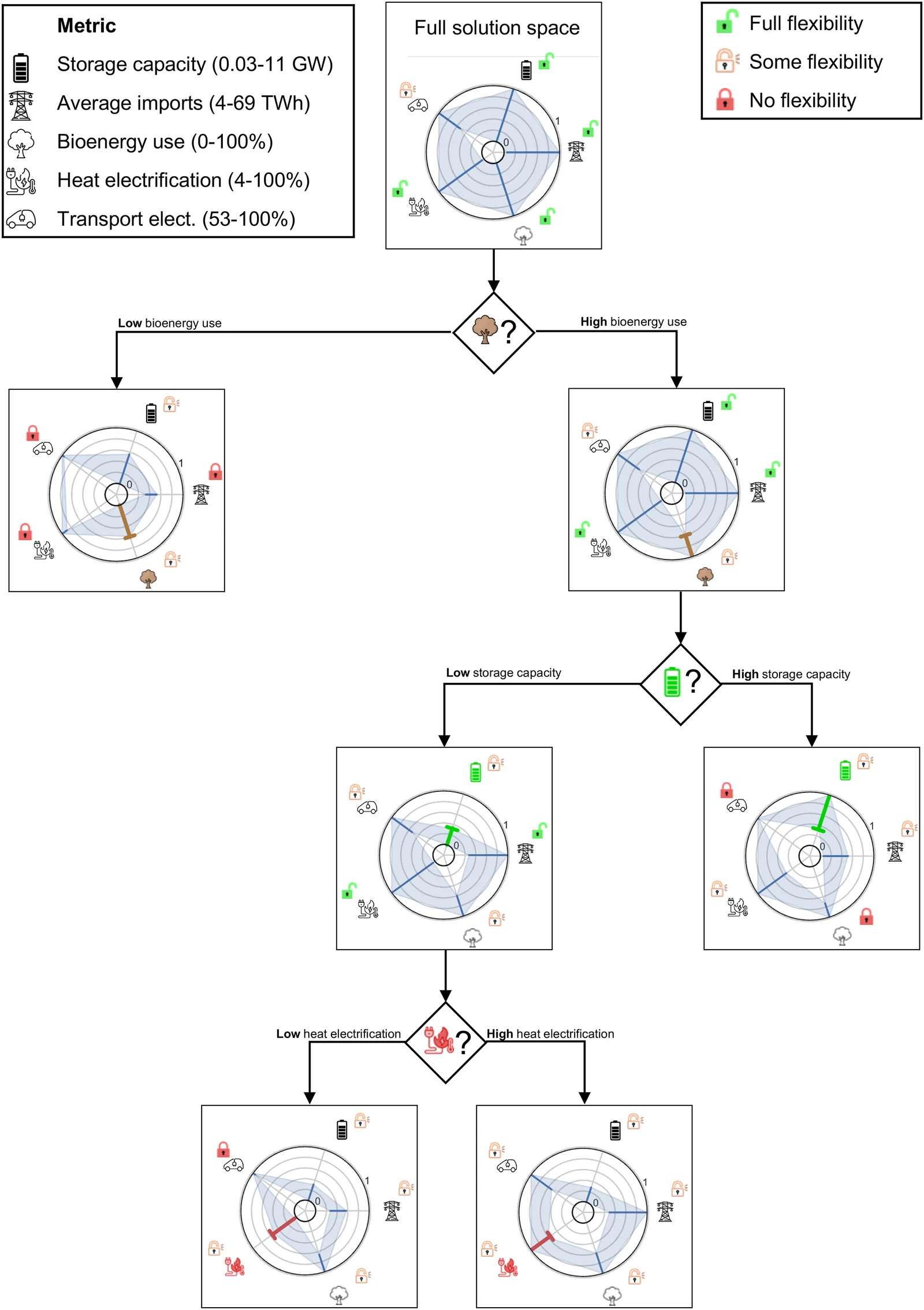
Decision tree for the European case study Decision tree breaking down the 441 alternatives for an energy self-sufficient, fossil-free European energy system presented by Pickering et al.^19^ into four storylines defined by three key drivers: bioenergy use, storage, and heat electrification For an explanation of the symbols, see Figure 2.

The high-bioenergy side of the tree retains the original freedom in all other outputs. Next, the tree splits the solution space on the installed storage capacity, which influences national imports: a low storage capacity is associated with medium-to-high imports, and vice versa. Thus, with combined high bioenergy and storage uptake, countries can limit their electricity import dependency. However, investing in storage capacity also leads to 100% transport electrification and high heat electrification to keep within the near-optimal cost range.

Overall, the decision tree clearly visualizes the two trade-offs between the five outputs of interest: First, bioenergy can be used either to avoid heat electrification or to avoid very high shares of transport electrification. Second, it highlights a trade-off between electricity imports and storage.

## Discussion

Our findings have key implications for modelers and decision-makers in the energy transition.

For modelers, considering uncertainty or exploring alternative solutions (e.g., via MGA methods) can lead to thousands of scenarios that can overwhelm decision-makers. Independently of how these scenarios are generated, the proposed method can help interpret results by breaking them down into a small number of storylines. These storylines are organized in a clear hierarchy by decision trees that split on the outputs of interest. While clustering of solutions is not new in the energy systems domain, this paper introduces the idea of training decision trees on outputs of interest corresponding to policy-relevant levers, which unveils how these levers are interlinked in the energy transition.

With respect to stakeholder engagement approaches, the decision trees can support discussions by illustrating the trade-offs between storylines. For example, in the European case study, we show that avoiding high electrification of the heating sector is only possible if most available bioenergy resources are allocated to energy use (Figure 4). We believe that these “to avoid A, you must accept B” statements reduce the danger of unrealistic feel-good storylines and move the discussion toward the real—potentially difficult—trade-offs that must be addressed. We do not suggest replacing the stakeholder engagement approach; on the contrary, the method begins with the definition of key outputs of interest, a step that should always involve stakeholders, and it can be applied iteratively in participatory settings. We assessed the interpretability of the storylines produced by our method in a workshop bringing together social scientists and modelers, with promising initial results that we report in the STAR Methods section and in the supplemental information.

Additionally, the presented method can streamline a major hurdle in large energy research consortia, where significant resources are often invested in agreeing on common assumptions and scenarios to align project outputs. The outcome of this process is a set of input assumptions and storylines that inform the different modeling teams. We propose a new paradigm to approach this initial phase by using decision trees to generate the storylines from a large set of modeling results. Moreover, explaining and streamlining the results of different models can help foster discussions between different modeling groups.

For decision-makers, we make energy scenarios more accessible by streamlining them into a limited set of storylines. By training decision trees on outputs of interest corresponding to policy-relevant levers, we show important trade-offs that can support the decision-making process. For example, in the global case study under uncertainty, while we build on results by Panos et al.,^18^ our findings are new: the decision tree visualizes how increasing sector coupling drives the switch from fossils to renewable energy (Figure 3). Additionally, we show that betting on continued fossil fuel usage only works if climate change proves unusually mild.

The European case study reveals that an energy-self-sufficient, fossil-free European energy system has some maneuvering space in the other considered metrics only if high bioenergy use is accepted. In other words, low use of bioenergy forces full electrification in all other sectors. Interestingly, this finding is mentioned as an example finding in the original study.^19^ The decision tree in Figure 4 systematically shows that bioenergy utilization is not just one of the many trade-offs, but it is *the* main driver splitting the solution space. Importantly, it shows that it is not possible to achieve climate neutrality in Europe at an affordable cost while having both a low bioenergy use and a low electrification in the heating and transport sectors.

### Limitations of the study

While our study has inherent limitations, these also highlight opportunities for further research and application. In the following, we outline these aspects together with possible directions for future work.

First, while defining the outputs of interest gives the flexibility to consider specific questions relevant to stakeholders, the decision trees will only visualize trade-offs between levers that are chosen as outputs of interest. This makes the choice of outputs a critical step that should always involve stakeholders. In the STAR Methods section, we propose a procedure to tailor the decision trees to specific questions that matter to stakeholders, which we did not test in this study and could be further validated in participatory workshops.

Second, as we use machine learning techniques, the outcomes are only as good as the underlying data and models, including their assumptions and constraints. We derived different insights from three recent peer-reviewed datasets: a subset of the AR6 scenario dataset,^16^ an uncertainty-driven global study^18^ and a near-optimal-solution-based European study.^19^ However, all these datasets and studies present limitations: The AR6 database is influenced by model fingerprints and a bias toward a few high-abundance models.^45^ In the global case study under uncertainty, the near-optimal solution space is not explored. Additionally, when applied to scenarios generated under uncertainty, decision trees highlight the options available today, each optimal under specific future conditions. However, the method does not assess the robustness of these alternatives—that is, what happens if an option chosen as optimal under some assumptions performs poorly under adverse future conditions. In the European case study, we do not know how uncertainty in input parameters affects the solutions, i.e., while all solutions are near-optimal for the nominal parameters, some might be more robust to uncertainty. An additional limitation of our case studies is that some technology options are not considered. While we show that decision trees give meaningful insights in all cases, and hence they could be applied to energy scenarios that are generated considering model uncertainty (e.g., via model ensembles), parametric uncertainty and near-optimal solutions, results cannot be fully immune against potential biases in the underlying data. Besides the adoption of debiasing procedures to address the uneven representation of models in model ensembles (as in our global case study), these concerns can additionally be mitigated by the adoption of transparency and openness practices in the modeling process, which are increasingly diffused in the energy modeling community.^72^^,^^73^ Additionally, addressing the robustness question would constitute a relevant extension of the proposed approach.

Third, our method requires as input a set of energy scenarios for a given region or country, which must first be produced if not already available. Depending on the scope of the analysis, different models can be used for this purpose, many of which are well-established, available as open source^25^^,^^74^ and can be adapted to the region or country of interest. The STAR Methods section presents different approaches to generate these scenarios and can be followed as a blueprint to apply the method to a new case study. In the STAR Methods section, we discuss the robustness of our findings with respect to the number of scenarios and with respect to the number of clusters used for training the decision trees, which together mitigate two other potential sources of bias in the underlying data.

Finally, while the proposed method can support the definition of energy and climate policies, our results do not directly translate into decisions or policy recommendations. Decisions can have causal effects that go beyond the correlations and interlinkages highlighted by our method, and which may be captured by sequential decision-making frameworks. Also, policy design is complex and requires inputs that may not be possible to capture with quantitative models or be readily available as model outputs. This can in part be mitigated by the selection of appropriate models, relevant outputs of interest, and possible model extensions to include additional policy indicators, which can be iterated with stakeholders. Such iteration and participatory engagement may not only improve the real-world applicability of the framework but could also foster a sense of ownership among policymakers, a factor shown to be critical for the successful implementation of energy scenarios.^52^ Future work may include participatory workshops with policymakers to validate the method’s usefulness in supporting real-world decision processes.

Overall, this paper proposes a method to support the communication between modelers, stakeholders and decision-makers, which is paramount for a swift and effective energy transition. The approach is scalable: it can be applied to global model ensembles as well as to local, city-level or industrial planning once scenario data are available and stakeholders agree on the outputs to track.

The decision trees combine quantitative information from hundreds of energy system scenarios with the intuitive interpretability of socio-economic storylines. In this way, decision-makers no longer need to choose between these two alternative approaches but can benefit from their respective added value. Our findings generate new insights by discovering structures in a dataset of energy system scenarios, irrespective of the way these scenarios are generated, and by systematically identifying multi-dimensional trade-offs. The main cautions are to use high-quality, transparently documented scenarios and to involve diverse actors when selecting the metrics, so that hidden model biases or narrow interests do not steer the tree. Done well, this method can offer a fast, intelligible, and inclusive compass for navigating the complex trade-offs in the energy transition.

## Resource availability

### Lead contact

Requests for further information and resources should be directed to and will be fulfilled by the lead contact, André Bardow (abardow@ethz.ch).

### Materials availability

This study did not generate new materials.

### Data and code availability

Further results from our case studies that support the findings of this article are presented in the Supplemental Information. The raw data used in the case studies and details on their preprocessing are available at: https://www.gitlab.ethz.ch/epse/systems-design-public/decide/. The software package is available at: https://www.gitlab.ethz.ch/epse/systems-design-public/decide/.

## Acknowledgments

F.B. and A.B. acknowledge funding by the Swiss Federal Office of Energy’s SWEET program as part of the project PATHFNDR. S.M. acknowledges support from the Swiss National Science Foundation under grant no. PZ00P2_202117 and from the European Union’s European Research Council (ERC), grant project no. 101164071 (UNITES). F.B. thanks Edith Baader for valuable discussions on decision trees. The authors thank the organizers and participants of the 4th “SSH (Social Science and Humanities) Energy Workshop” (Zurich, June 17–18, 2024) for the valuable feedback.

## Author contributions

F.B.: conceptualization; methodology; software; data curation; investigation; writing – original draft; visualization. S.M.: conceptualization; methodology; software; investigation; writing – original draft; funding acquisition. W.W.: conceptualization; methodology, writing – review and editing. I.S.: conceptualization; methodology, writing – review and editing. A.B: conceptualization; methodology, writing – review and editing; funding acquisition.

## Declaration of interests

A.B. served on review committees for research and development at ExxonMobil and TotalEnergies. A.B., S.M., and I.S. have ownership interests in firms that render services to industry, some of which may provide energy planning services. All other authors have no competing financial interests.

## Declaration of generative AI and AI-assisted technologies in the writing process

During the preparation of this work, the author(s) used ChatGPT and Claude in order to improve the readability and language of the manuscript. After using this tool/service, the authors reviewed and edited the content as needed and take full responsibility for the content of the published article.

## STAR★Methods

### Key resources table

**Table:** 

REAGENT or RESOURCE	SOURCE	IDENTIFIER
**Depositeddata**		

AR6 Scenario Database	https://zenodo.org/records/7197970	
Data for the global decarbonization pathways under uncertainty case study	https://zenodo.org/records/8045287	
Data for the European energy transition case study	https://zenodo.org/records/6546817	

**Softwareandalgorithms**		

Python	https://www.python.org	

### Experimental model and study participant details

#### Human participants

Our participatory modeling workshop (see method details) took place as part of the 4th “Social Science and Humanities (SSH) Energy workshop”, which took place in Zurich (Switzerland) on June 17–18, 2024, involving around 50 participants, socio-economic scientists and energy modelers working on energy transition policies. Of these, 13 participants took part in our exercise and were divided into four groups to derive storylines from the decision trees. The study involving human participants has been approved by the ETH Zurich Ethics Commission (approval reference EK-2024-E−27) and informed consent was obtained from all participants.

### Method details

We first summarize how the scenarios used as inputs to our analyses are typically generated. Note that, throughout the paper, we use the term *scenario* to refer to a model realization described by a set of outputs, such as an energy system configuration in the case of an energy model. We propose a systematic procedure to choose appropriate outputs of interest depending on the questions, and discuss its application to the Global and European case studies. Then, we present the method to derive the decision trees. The software package to reproduce our results and apply the method to other case studies is available open-source at: https://www.gitlab.ethz.ch/epse/systems-design-public/decide/. Overall, the different sections constitute a blueprint that can be followed to apply our method to different case studies. A detailed list of the steps is reported in the supplemental information.

#### Preliminaries

The energy scenarios, which are the basis for our decision trees, are typically generated from solving a mathematical optimization problem. An optimization problem consists of an objective function *f*, typically a cost function, which is minimized over the vector of decision variables **x** (e.g., the investment and operation strategy) subject to constraints **c** (e.g., resource availabilities or meeting demand):

(Equation 1) $$\begin{array}{ll} & \underset{x}{\text{min}}\;\;f\left(x,\theta \right)\\ & \text{s.t.}\;\;c\left(x,\theta \right)\leq 0 \end{array}$$

In Problem 1, both the objective function *f* and the constraints **c** depend on parameters ***θ***. These parameters ***θ*** are input data such as prices or demands. Solving an optimization problem results in one optimal decision vector **x**∗. However, as discussed in the literature review, a “single optimal solution” might not approximate real-world energy scenarios well, and hence two methods are typically used to generate alternative solutions. The first method considers uncertainty, i.e., accounts for the fact that the values of the parameters ***θ*** (such as the future gas prices or energy demands) are unknown. Given a probability distribution for the uncertain parameter vector ***θ***, sampling – often, Monte Carlo sampling – is performed to obtain *N* possible realizations of the uncertain parameter vector ***θ***. For instance, Panos et al.^18^ create *N* = 1000 plausible realizations of ***θ***. These *N* parameter vectors ***θ***_*i*_, *i* = 1, *…*, *N* lead to *N* optimal decision vectors $x_{i}^{*}$, which are the inputs to our global case study under uncertainty.

The second method, modeling to generate alternatives (MGA), explores the space of near-optimal solutions. Near-optimality is defined by an acceptable cost overshoot *ϵ*, e.g., 10% in the study of Pickering et al.^19^ Thus, all solutions **x**_*i*_ with an objective function

(Equation 2) $$f\left(x_{i},\theta \right)\leq \left(1+\varepsilon \right)f\left(x^{*},\theta \right)$$

are accepted as near-optimal.

Concretely, MGA studies solve a sequence of optimization problems with the objective of generating maximally different solutions **x**_*i*_. To this end, some key decision variables are minimized or maximized subject to the near-optimality constraint in Equation 2. For example, Pickering et al.^19^ follow this approach to generate 441 near-optimal solutions for the European energy system, which are the inputs to our European case study. The MGA method presented in,^19^ SPORES, can be used to generate a similar set of results for other countries or regions.

Overall, both uncertainty sampling and MGA generate many plausible decision vectors **x**_*i*_ and, thus, devise possible scenarios for the energy transition. In the next subsections, we describe how our method reduces the large number of possible decision vectors **x**_*i*_ to an interpretable decision tree. Note that, although the studies in this work consider either uncertainty sampling or MGA, uncertainty sampling and MGA could also be applied in combination. While the minimum number of scenarios may vary depending on the case study, based on our numerical experiments (reported in the following), we recommend generating a few hundred scenarios to ensure the convergence of the splits generated by the decision trees.

#### Selecting outputs of interest

The method starts from the definition of *m* outputs of interest **y**, which can be calculated as functions of the decision variables, i.e., **y** = **h**(**x**). This step is necessary, as the vector of decision variables **x** typically consists of thousands of outputs, while only a few high-level outputs are needed to inform decision-makers. In Figure 5 we present a systematic approach to help identify the outputs of interest engaging both modelers and stakeholders. In this section, we show how we applied this approach to the case studies reported in the paper. Note that this is an illustrative application, as no dedicated stakeholder elicitation exercise was conducted to identify the outputs of interest in this study.

**Figure 5:**
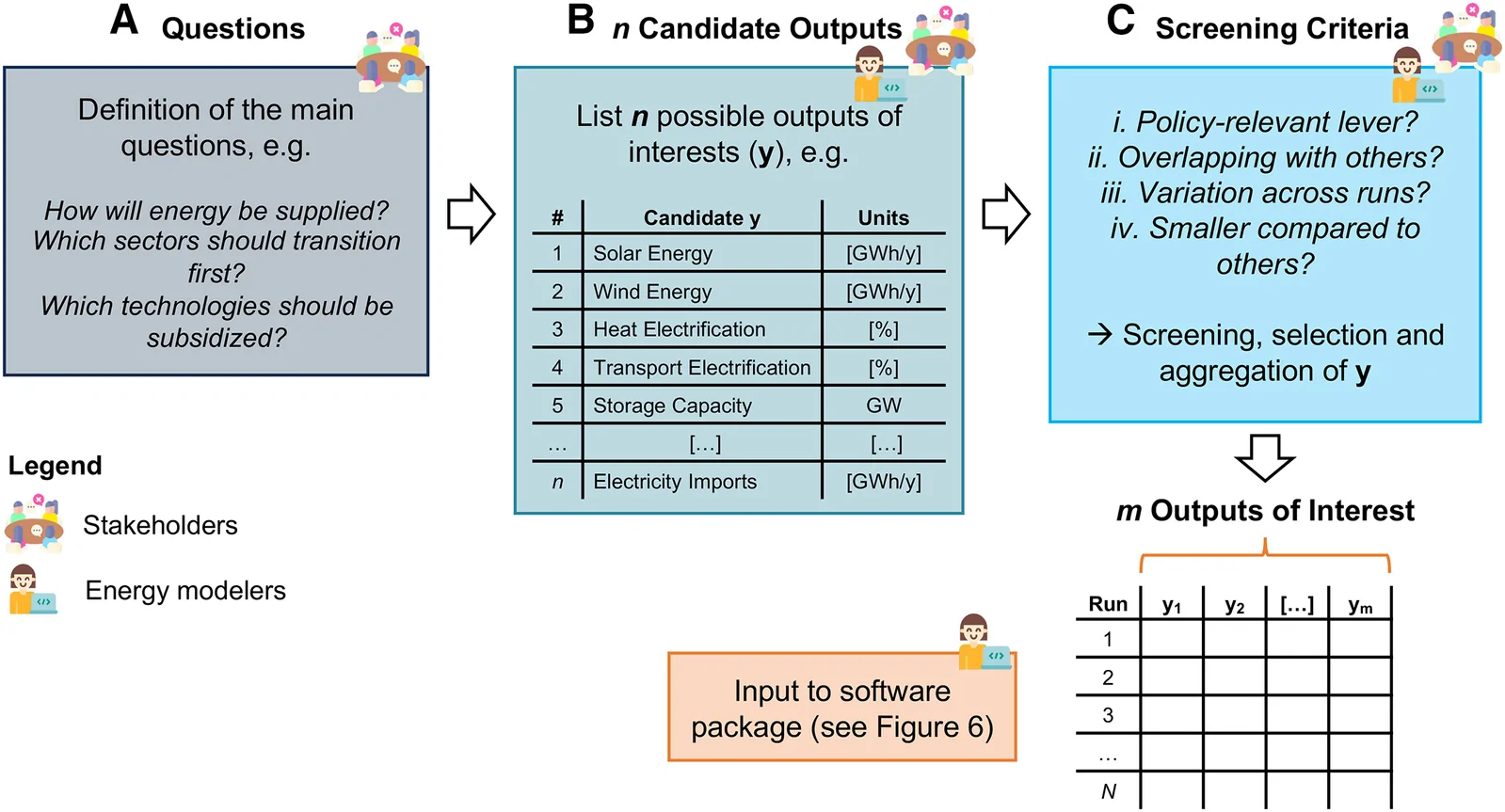
Systematic approach to identify outputs of interest **y** engaging both modelers and stakeholders First, relevant questions are identified by stakeholders. (A) Then, stakeholders and modelers list *n* (normally 10 to 20) candidate outputs of interest that can address the identified questions. (B) Finally, screening criteria are applied to reduce the *n* candidates to *m* (normally 3 to 7) outputs of interest **y**. (C) The process results in a *N* × *m* matrix of scenarios, which serves as input to our software package.

Step (A) concerns the definition of the key questions, which are identified by stakeholders based on their priorities. For example, stakeholders may be interested in understanding the role played by different renewable resources in the energy transition, which technologies should be subsidized or the extent to which future energy systems will rely on imports from neighboring countries. In Step (B), modelers and stakeholders identify a list of *n* possible outputs of interest that can address the identified questions. For example, if the question concerns the role played by the different renewable resources in the energy transition, the yearly electricity production from wind and solar energy are meaningful outputs of interest. In Step (C), a screening process reduces these *n* (normally 10 to 20) candidates to *m* (normally 3 to 7) outputs of interest **y**. The first criterion is choosing outputs **y** that correspond to policy-relevant levers: For example, if decision-makers can opt to support electric mobility with subsidies or by banning fossil cars, then the share of electric mobility is a meaningful output. Second, outputs that do not vary across the *N* runs are discarded. Third, outputs that are small with respect to other outputs or that are overlapping can be aggregated. Note that to allow comparison of outputs, it is important to express the outputs of interest in the same units whenever possible. In the supplemental information, we additionally show how meaningful outputs of interest are connected by mass and energy balances. This systematic process finally results in *m* outputs of interest and a *N* × *m* matrix of scenarios which serves as input to our software package.

For the global case studies, we focus on two questions: (i) How is primary energy supplied and (ii) how are the sectors industry, heating and transport transitioned. As *n* candidate outputs of interest, we consider the 39 outputs reported in the study by Panos et al.,^18^ to which we apply the screening criteria (Step (c) in Figure 5). First, six outputs can be excluded because they are not of interest for our questions (e.g., the total system cost) or because they do not correspond to actionable decisions (e.g., the total final energy consumption). For the first question, primary energy supply, we aggregate the contribution from renewables in one output of interest, i.e., (1) global primary energy supply by renewable energy, varying between 278 and 1119 EJ/y. As a second output of interest, we consider (2) the global primary energy supply by fossil fuels, which also shows a significant variation across the runs (238–1279 EJ/y). We do not consider nuclear energy, as it only varies between 6.6–7.1 EJ/y. Similarly, we exclude outputs related to the production and use of electricity and hydrogen, because they are secondary energy carriers not directly entering the primary energy balance. For the second question, concerning sector coupling, we focus on the transition in the three main non-electricity sectors. In this case, output metrics are expressed as relative shares in Panos et al.,^18^ which we convert into annual energy flows. This leads us to consider transport, heating and industry as the main sectors, and to the exclusion of cooking, which represents a minor share in terms of energy flows. Aggregating the remaining outputs leads to the final three outputs of interest for sector coupling: electrification of industry (varying between 16 and 71%), electrification of residential heating (14–89%), and the share of non-fossil transport, i.e., based on electricity, biofuels or hydrogen (46–87%).

For the European case study, Pickering et al.^19^ report nine outputs in their analysis, which we consider as our *n* candidate outputs of interest (Step (b) in Figure 5). Out of these nine outputs of interest, four are excluded because they do not correspond to policy-relevant levers. Specifically, we exclude two Gini coefficients and a Pearson correlation coefficient as these coefficients are hard to interpret for non-experts and also not directly possible for decision-makers to influence. Moreover, we do not consider the curtailment of renewables, as the maximum curtailment considered in the study is 6%. We argue that if a curtailment of renewable energy in this order of magnitude is cost-efficient, it will not lead to public discussions. This leads to the five outputs of interest addressing the questions: (1) bioenergy use, (2) share of heat electrification, (3) share of transport electrification, (4) average electricity imports, and (5) installed storage capacity.

#### Generating decision trees

After selecting the outputs of interest **y**, decision trees are generated in three steps: (1) clustering, (2) training a decision tree, and (3) reordering the data points (Figure 6). We chose decision trees over other machine learning methods providing similar information, e.g., the Rule Lists method,^75^ as they are very popular ML models due to (i) their interpretability and (ii) their generally high accuracy across a broad range of domains.^76^ Additionally, the choice is motivated by the high familiarity that stakeholders have with decision trees, which is documented in the “scenario discovery” literature.^66^ Note that, in the following, we represent energy scenarios purely by their outputs of interest **y**. That is, the *N* decision vectors **x**_*i*_(*i* = 1, *…*, *N*) generated by uncertainty sampling and/or MGA are converted to *N* output of interest vectors **y**_*i*_ that define the corresponding energy system scenarios.

**Figure 6:**
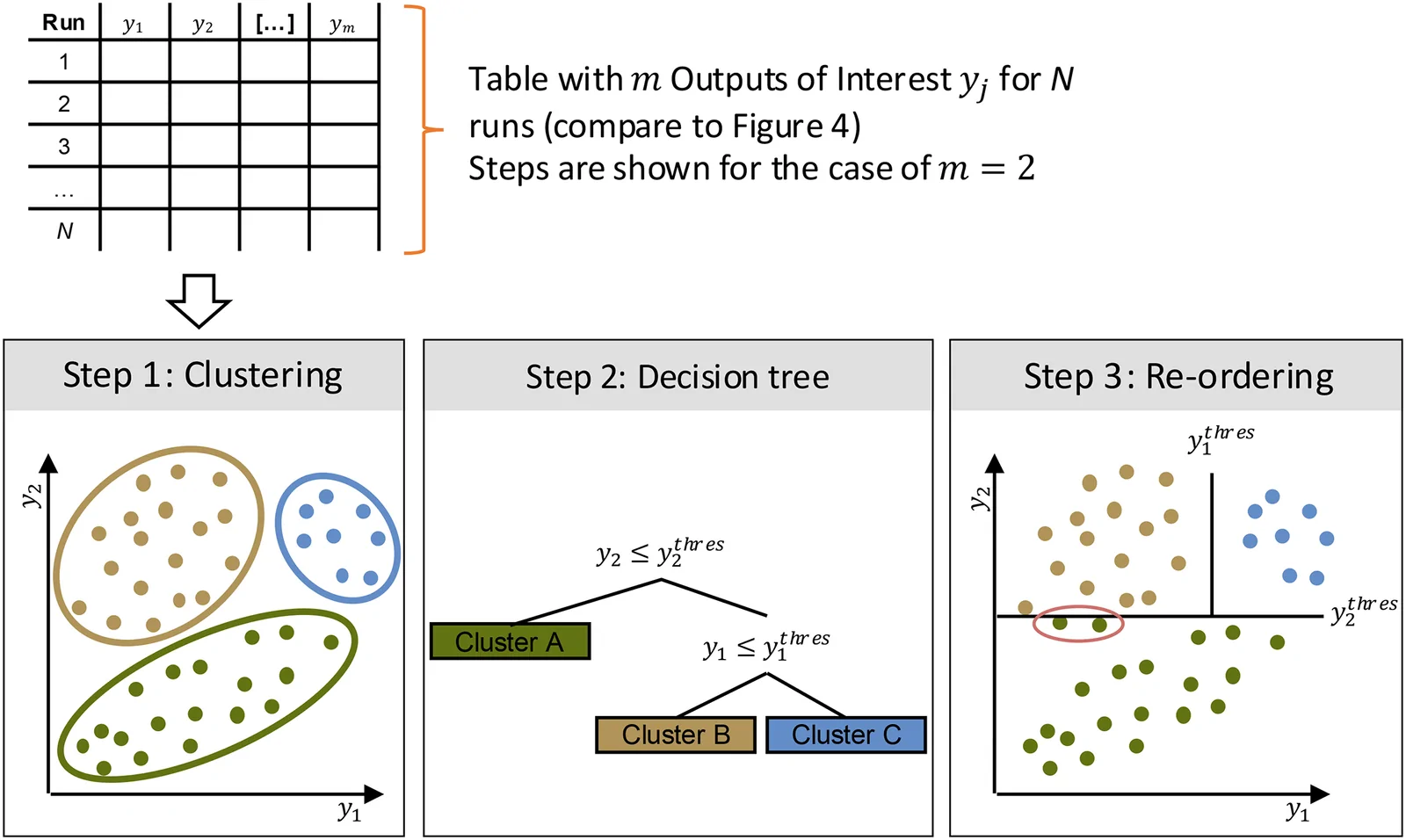
Conceptual example of the procedure for generating the decision trees, using only two outputs of interest: *y*_1_ and *y*_2_, i.e., *m* = 2 In step 1, *k*-means clustering splits the data points into three clusters. In step 2, a decision tree is trained to predict the cluster based on the outputs of interest **y**. In step 3, the points are reordered according to the splits learned by the tree such that a small number of points indicated with a red circle are re-assigned to a different cluster. The re-assignment is necessary as decision trees always split on a single variable, thus giving orthogonal splits.

In Step 1, we group the *N* different scenarios into *k* clusters, where *k* ≪ *N*. We describe how to choose *k* in the next section. Clustering is carried out using the *k*-means algorithm,^76^ which has shown the best performance in the scenario discovery study by Jafino and Kwakkel^70^ and was used in the recent MGA study by Prina et al.^65^ More specifically, we use the Python package sklearn.cluster.KMeans with default settings. The *k*-means algorithm groups a set of data points, here the *N* solutions represented by the output vectors of interest **y**_*i*_, to *k* clusters, where *k* is pre-defined. The rationale is to group similar points by minimizing a distance measure. With the standard settings, the distance measure is defined as the sum of the squared distances of each point from the cluster center. In mathematical terms, clustering aims to find *k* cluster centers $y_{c}^{\text{center}}$ and a mapping *K* from {1, *…*, *N*} to {1, *…*, *k*} such that the distance

(Equation 3) $$d=\overset{N}{\underset{i=1}{\sum }}{\left\|y_{i}-y_{K\left(i\right)}^{\text{center}}\right\|}_{2}^{2}$$

is minimised. The result of the clustering is that every energy system scenario **y**_*i*_ is assigned to a cluster *c* = 1, …, *k*.

In Step 2, we interpret the clusters and identify the key drivers by training a decision tree.^76^ Decision trees are a supervised machine learning method and hence are trained on labeled data. In our case, the labeled data are the *N* energy system scenarios **y**_*i*_, and the labels are the cluster numbers *c*_*i*_ assigned to the data points by the previous clustering step. The decision tree learns to predict the label *c*_*i*_ given the energy system scenario **y**_*i*_. For this prediction, the tree applies binary splits to the dataset, i.e., one dimension *y*_*j*_ and a threshold $y_{j}^{\text{thres}}$ are chosen, and the data points are split into points with $y_{j}\leq y_{j}^{\text{thres}}$ and points with $y_{j}>y_{j}^{\text{thres}}$. At every node of the tree, the split is chosen that minimizes the impurity of the data points. The impurity quantifies the heterogeneity of the points within a node of the tree, as set out mathematically in Hastie et al.^76^: If a node of the tree only consists of points that belong to a single cluster, the impurity is zero as this node of the tree is perfectly ordered. On the contrary, if the number of points belonging to a cluster is the same for all clusters, the impurity is at its maximum value. We limit the number of leaves of the tree to the number of clusters *k*. In our numerical experiments, a tree with a number of leaves equal to the number of clusters has high prediction accuracy, i.e., 99% of the data points are correctly assigned to their clusters. To implement the decision trees, we use the Python package sklearn.tree.DecisionTreeClassifier with default settings, using “entropy” as the splitting criterion to maximize the homogeneity of scenarios within each leaf.

Finally, in Step 3, we reorder the energy scenarios **y**_*i*_ into *k* clusters according to the rules of the decision tree: As illustrated in Figure 6, the few points that are predicted wrongly by the decision tree are re-assigned to the other clusters accordingly. With this re-assignment, the clustering becomes interpretable: Before the reordering, a cluster is simply a set of *m*-dimensional scenarios **y**_*i*_, where *m* is the number of outputs of interest. After the reordering, a cluster is defined by the splits of the tree that lead to this cluster, e.g., Cluster B in Figure 6 is defined by the two conditions $y_{2}\geq y_{2}^{\text{thres}}$ and $y_{1}\leq y_{1}^{\text{thres}}$. In our numerical experiments, this reordering of points does not significantly affect the quality of the clustering. As shown in the supplemental information, the distance measure *d* (Equation 3) only increases by 0.3% (global case study under uncertainty) and 1% (European case study). If the decrease in clustering performance due to the reordering in the combined clustering plus decision tree approach is too high for other case studies, interpretable clustering via optimal trees could be used,^77^ which combines clustering and decision tree generation into a mixed-integer optimization problem. As these optimal trees can have a high computational cost, we use a pragmatic reordering approach, which in our experiments enjoys high accuracy with low computational time.

#### Choosing the number of clusters *k*

Finally, we discuss the choice of the number of clusters *k*. To identify a reasonable value for *k*, we use the elbow method,^78^ which plots the distance measure *d* vs. the number of clusters *k*. Typically, the distance measure decreases rapidly for small numbers of clusters. For a higher number of clusters, the distance measure only decreases slowly. Ideally, a so-called elbow point should be chosen as *k*, located where the distance measure is already small, and adding additional clusters does not substantially further reduce the distance measure.

In this work, the elbow serves as an upper bound. Still, it can be reasonable to choose a smaller number of clusters to ease communication. As the human brain has capacity limits,^14^^,^^79^ it is advised for effective communication not to present more than five alternatives.^80^^,^^81^ Moreover, the number of storylines presented in socio-economic research is typically between two and five.^48^ Thus, it would be desirable from a communication point of view to have five or fewer clusters.

As a third criterion for choosing the number of clusters, we analyze the splits of the decision tree: If the tree splits on the same output several times, the clusters can become less meaningful. For example, in the European case study, we choose *k* = 4 which is below the elbow (Figure S10). With these 4 clusters, we observe 3 splits in the outputs: bioenergy use, storage capacity, and heat electrification. With 5 clusters, an additional split on bioenergy use appears that does not add substantial insights, as shown in Figure S12. Thus, once the decision tree starts to repetitively split on the same outputs of interest, it can be reasonable to reduce the number of clusters even if this number is below the elbow. Similarly, in the global case study under uncertainty, we choose 3 clusters as the elbow curve shows a clear change in slope at *k* = 3 (Figure S1). Additionally, we observe that by setting *k* > 3 clusters, the decision tree starts to split on the same output of interest multiple times (Figure S2). Specifically, with 4 clusters, the tree differentiates between low, medium, and high industrial electrification instead of only high and low. In our view, this additional differentiation increases the total number of clusters and hence makes communication more challenging without adding much high-level insight. For reference, the decision tree with a fourth cluster is shown in Figure S3.

Finally, we visualize the resulting clusters and decision tree by showing the splits and representing every branch and leaf of the tree with a radar plot showing the space covered with respect to the outputs of interest **y**, as visualized in Figures 2, 3, and 4.

#### Participatory modeling workshop

As discussed above, our decision trees can help define common storylines to align research outputs in large consortia where multi-model comparisons are performed. In current practice, storylines are first developed in dedicated workshops, which are then translated into assumptions by the different modeling teams. This process is often affected by potential biases and may not explore relevant storylines. In addition, it requires significant effort and often stretches far into the project timeline. To address these issues, our paper proposes a new paradigm: first, we systematically explore the decision space with one (or more) models; then, we run our method to obtain a decision tree and propose potential storylines. An open question remains on how well our storylines can be translated into the descriptive storylines typically used in large research consortia. We tested this research hypothesis in a dedicated workshop as part of the 4th “Social Science and Humanities (SSH) Energy workshop” (see experimental model and study participant details for participant information). We asked participants to derive storylines from the four energy strategies identified by the European decision tree (Figure 4). The workshop abstract and program are reported in the supplemental information.

The 13 participants were divided into groups to derive storylines from the decision trees. An example output of the group focusing on Storyline D, characterized by a high use of bioenergy and a high deployment of storage (Figure 4), is reported in Figure S18, together with a “policy speech” for the same storyline, which was emailed by a workshop participant after the workshop.

### Quantification and statistical analysis

In this section, we assess the robustness of the tree obtained for our global case study under uncertainty with respect to the number of input scenarios. In the main text, we report the decision tree using the full dataset of 1000 scenarios (Figure 3). Here, we assess how the first and second splits of the resulting decision tree change as we vary the number of input scenarios. To do this, we run our algorithm for different subsets of scenarios (containing 25, 50, 100, 200, 400, and 800 scenarios, respectively), and, for each sample size, we repeat the random sampling 50 times to account for stochasticity. The results are reported in Figure S4: after reaching sample size 100, the tree splits in 97.5% of the cases on the same features as the full model using 1000 scenarios, namely the feature “Electric Heating” (SEH, which is most often the first split) and on “Electric Industry” (SEI, which is most often the second split).

While this shows that the tree splits converge at a low number of scenarios, we further analyzed the alternation of the first and second splits between these two features. This is visually represented in Figure S5, in which “Electric Industry” is the first split and “Electric Heating” is the second split, which can be compared to Figure 3, where the splits are inverted. The comparison shows that the three final clusters (indicated in red, green, and blue) obtained in both cases are identical, i.e., inverting the first and second split between “Electric Industry” and “Electric Heating” results in the same trade-offs. This is due to the fact that in the global dataset, a high electrification of industry implies a high electrification of heating, but a low electrification of industry does not allow us to conclude anything about the electrification of heating. In summary, this analysis shows that, for the global case study under uncertainty, results are robust even with low numbers of scenarios and do not change for a scenario size greater than 100, which we recommend as a minimum sample size for the application of our method.
