# Supplementary material for: Seeing the forest for the scenarios: Using decision trees to draw conclusions from large energy scenario sets
Source: iScience. 2026 Sep 30;29(10):117079. doi: 10.1016/j.isci.2026.117079 (PMC13657154; doi:10.1016/j.isci.2026.117079)
Supplement: Supplementary file 1 [file mmc1.pdf]

iScience, Volume *29*

Supplemental information

Seeing the forest for the scenarios: Using decision trees to draw conclusions from large energy scenario sets

Florian Baader, Stefano Moret, Wolfram Wiesemann, Iain Staffell, and André Bardow

1 Blueprint for the application of our method

This section complements the Methods section in the main paper by summarising the steps needed to apply our method to other case studies.

1. Are more than 100 scenarios available for the case study of interest? If yes, skip to point 4.

2. Create a model for the case study of interest. Depending on the scope of the analysis, different models can be used for this purpose, many of which are well-established, available open-source [1, 2] and can be adapted to the region or country of interest.

3. Generate at least 100 scenarios for the case study of interest. The section "Preliminaries" in the main paper indicates how to generate scenarios under parametric uncertainty or to explore the near-optimal space using modeling to generate alternatives (MGA).

4. Define and select outputs of interest, possibly iterating with stakeholders, as indicated in the section "Selecting outputs of interest" in the main paper.

5. Use our open source tool DECIDE (https://www.gitlab.ethz.ch/epse/systems-design-public/decide/) to generate the decision trees. Additional details are provided in the section "Generating decision trees" in the main paper.

6. Assess the robustness of the obtained tree with respect to the number of clusters and to the number of scenarios, as described in the next sections.

2 Global case study under uncertainty

In this section, we provide additional information regarding the global case study under uncertainty using data from [3]. Specifically, we discuss the uncertainty analysis (Subsection 2.1). Finally, in Subsection 2.2, we extend the analysis in the main paper to the full dataset in [3].

2.1 Analyzing uncertainty by scenario discovery

To analyze which of the 18 uncertain parameters considered by Panos et al. [3] are the main drivers leading to the 3 clusters we identified, we use the scenario discovery method proposed by Gerst et al. [4]. In scenario discovery, a classification tree is trained to predict the cluster c. In contrast to our approach, where we train a classification tree to predict the cluster c as a function of the outputs of interest $\mathbf{y}$, the scenario discovery approach trains a classification tree to predict the cluster c as a function of the uncertain inputs $\boldsymbol{\theta}$.

In principle, a classification tree can be trained that perfectly predicts the data; however, this decision tree will typically have a high number of leaves, making it hard to interpret. Moreover, a decision tree with a high number of leaves might overfit. Thus, Gerst et al. [4] propose to limit the number of tree leaves as a compromise between an interpretability and a coverage score. The interpretability score is defined as one over the number of variables that the classification tree branches on. Coverage is defined as the fraction of points that the tree correctly assigns to their clusters. This coverage is additionally calculated in a cross-validation to avoid overfitting. Here, we use a 5-fold cross-validation.

Analyzing interpretability vs. coverage for our case study, we observe a plateau at an interpretability of 0.5 up to 17 tree leaves (Figure S6). This means that a classification tree that is allowed to make up to 16 splits of the dataset only uses 2 of the 18 uncertain parameters. With these 2 uncertain parameters, a cross-validation coverage of 87% is achieved (Figure S6), i.e., for 87% of the scenarios, the correct cluster is predicted based on only 2 of the uncertain input parameters. These 2 parameters are climate sensitivity and demand elasticity, discussed in the main paper. With more than 17 tree leaves, the interpretability score drops without substantial increase in the cross-validation coverage. Thus, we choose a classification tree that only branches on 2 of the 18 uncertain input parameters as it is also more easily accessible than a classification tree with 17 leaf nodes. Additionally, having only two parameters allows for representing the solution space in a 2-D plot. The result is shown in Figure 3 (b) of the main paper, where we plot demand elasticity over climate sensitivity for the 1000 Monte Carlo runs. The 1000 points are colored according to the cluster they belong to in Figure 3 (a), revealing that scenarios with high penetration of fossils and low sector coupling assume low climate sensitivity and low demand elasticity.

2.2 Considering varying climate targets

In the previous analysis and in the main paper, we considered 1000 scenarios from Panos et al. [3] with the target not to exceed a 2℃ temperature increase by the end of the century. However, Panos et al. [3] study the global energy transition with three different variants:

1. 2C_SSP2: This variant sets the 2℃ target at the end of the century. This is the variant used in the main paper.
2. 2C_SSP2_DA30: This variant sets the 2℃ target at the end of the century but delays any climate action after 2030.
3. 1p5c_OS_SSP2: This variant sets the 1.5℃ target at the end of the century.

For every variant, 1000 scenarios are generated. In this section, we consider all three variants simultaneously, i.e., 3000 scenarios in total, to study the effect of the different variants on the resulting storylines. Note that Panos et al. [3] perform an additional 1000 runs considering the business as usual scenario without any climate action, which we do not include here.

First, we observe that the ranges of variations with respect to the 5 outputs of interest do not vary substantially if including all 3 variants (Table S1). Again, we use $k = 3$ clusters as the elbow curve shows a nearly identical curve compared to the previous one (compare Figure S1 to Figure S7). Only the distance measure d is roughly higher by a factor of 3, which is reasonable, as the number of scenarios increases from 1000 to 3000.

The resulting decision tree shows the same splits in a different order: First, the tree splits on industry electrification and only second on heat electrification (see Figure S9 (a), compared to Figure 3 (a) in the main paper). Still, the resulting storylines are the same, as high industrial electrification implies high heat electrification (Figure S9 (a)), and low heat electrification implies low industry electrification (Figure 3 (a) in the main paper).

As in Subsection 2.1, we analyze the underlying uncertainties associating the Monte Carlo scenarios with the identified storylines. However, here, there are 19 instead of 18 uncertain inputs, as the variant constitutes a 19th uncertainty. A decision tree splitting on only 3 of these 19 inputs achieves a cross-validation coverage of 88% (Figure S8, compare to Figure S6). These 3 inputs are (i) the variant and – as in the main paper – (ii) climate sensitivity and (iii) demand elasticity. For all the three variants, we observe the same trend, i.e., betting on fossils is betting on low climate sensitivity and low demand elasticity (Figure S9 (b)). However, the border between the three storylines shifts between the variants. For example, if the red storyline with a high penetration of fossils is chosen, the input space in which the 1.5℃ target can be reached is much smaller (Figure S9 (b)). Consequently, the storylines with high shares of fossils and low sector transition are even riskier if one wants to achieve the 1.5℃ target.

3 European case study

In this section, we provide additional information regarding the European case study. In Subsection 3.1, as introduced in the main paper, we show how a quantitative analysis of energy balances can help choose the outputs of interest. As the original energy scenarios were created without an uncertainty sampling, the uncertainty analysis shown in Subsection 2.1 cannot be applied here.

3.1 Decision tree from a systematic screening of balances

As discussed in the Methods section of the main paper, defining the outputs of interest is always, to some degree, subjective as it reflects the stakeholder's interest. Still, choosing the outputs of interest can also be done more systematically based on the observation that interesting trade-offs occur between outputs of interest that are connected by mass or energy balances. This is because changing one entry in a balance always causes at least a second change. Thus, outputs of interest can be derived systematically by screening the varying parts of balances. In this section, we show this systematic identification for the European case study, where all energy flows were published [5]. To this end, we consider all flows in the overall energy and electricity balance, and train a decision tree on the flows with the highest range of variation. We choose the overall energy balance as it shows differences in how energy is supplied, and the electricity balance as electrification is the most important measure for sector coupling. The two balances and the considered flows are shown in Figure S13.

The four flows with the highest range over the 441 scenarios are: power to synthetic fuels, renewable electricity, bioenergy, and power to heat, all having a range greater than 2 PWh, while the next energy flow only shows a range of 0.8 PWh (Figure S14). Note that the flow "power to synthetic fuels" includes power used for electrolysis, hydrogen to methanol processes, hydrogen to liquid processes, and direct air capture providing carbon feedstocks for synthetic fuels.

We choose $k = 5$ clusters, as suggested by the elbow curve (Figure S15) even if this leads to 2 splits on the same output of interest, which we avoided in the previous case analysis (Figure S16). The reason is that with 5 clusters the tree splits on the power-to-heat flow for the first time, which shows a trade-off that would not be present in a tree with only 3 clusters. The resulting decision tree is depicted in Figure S17. As in the main paper, the first split is on bioenergy and shows that a low bioenergy utilization shrinks the space in all other outputs of interest.

For high bioenergy use, the following split is on the deployment of renewables. Lower deployments of renewables is associated with medium power-to-heat and low power-to-synthethic-fuel flows. The next split is on power-to-heat deployment. This split also determines the range of the power-to-synthethic-

fuel flow.

This section demonstrates that outputs of interest can be chosen systematically by screening balances. To use this systematic approach, stakeholders would not choose the outputs of interest directly but instead indicate their domain of interest, e.g., additional electricity demands caused by sector coupling. Based on these inputs, modelers can choose the balance to screen – the electricity balance in this case – and identify the flows with the largest range. Using these flows as outputs of interest results in decision trees demonstrating interesting trade-offs.

4 Participatory modeling workshop

4.1 Workshop description

Title: Building Narratives from Complex Energy Model Outputs: A Participatory Modelling Exercise.

Facilitators: Stefano Moret and Gabriel Wiest, ETH Zurich.

Abstract: Energy system models can help to identify and quantify energy transition pathways. In this workshop, we explore if/how we can use these models to systematically derive narratives and storylines commonly used in the SSH domain to support decision-making. Specifically, we present a machine learning method to translate large sets of energy transition scenarios, which are often challenging for policymakers to interpret and act upon, into a small set of actionable and interpretable options. After introducing our method, we will focus on a case study where the method streamlines hundreds of options to reach net-zero into four transition pathways on the decision-makers' table. Participants will learn about energy models and their applications; then, as a participatory modelling exercise, they will create narratives for these four pathways, which will be discussed to evaluate the effectiveness of our method in guiding decision-makers to prioritize the key factors that will shape the energy transition.

Program: The workshop lasted 90 minutes, structured as follows:

- Minutes 0-30: 20 minute presentation by the facilitators to introduce the method and the activities, followed by 10 min. Q&A.
- Minutes 30-70: The 13 participants were divided into four groups. Each group was assigned a different storyline of the European decision tree (Figure 4 in the main paper), with the task of creating a descriptive storyline, similar to the storylines typically used in large research consortia (examples were provided) using the flipchart template shown in Figure S18.
- Minutes 70-90: Each group presented their storyline to the other groups, and provided feedback on the method.

4.2 Description of Storyline D

As an example of the output that can be produced starting from our decision trees, a workshop participant produced a "policy speech" for Storyline D, which we report below.

Title: A Green Vision for Europe: Transforming Our Future

Introduction: As the European Union faces the urgent challenge of the climate crisis, it is imperative to embrace a bold and transformative vision for a sustainable future. Our political program for the Green Transition outlines a comprehensive strategy to lead Europe towards environmental stewardship and economic resilience. By focusing on high storage capacity, significant bioenergy utilization, extensive transport electrification, and strategic heat electrification, while maintaining moderate imports, we aim to create a sustainable and self-reliant Europe. This vision will harness the power of innovation and collaboration to ensure a greener, more prosperous future for all European citizens.

High storage capacity: At the heart of our green transition strategy is the development of high storage capacity for renewable energy. Effective energy storage is crucial for managing the variability of renewable sources such as wind and solar power. By investing in cutting-edge battery technologies, pumped hydro storage, and other innovative storage solutions, we will ensure a reliable and consistent energy supply. These technologies will allow us to capture and store excess energy during periods of high production, making it available when demand peaks. This approach will not only stabilize our energy grid but also reduce our reliance on fossil fuels and enhance energy security across Europe.

High bioenergy usage: Bioenergy plays a pivotal role in our vision for a sustainable Europe. By utilizing organic materials such as agricultural residues, forestry by-products, and dedicated energy crops, we can generate renewable energy while addressing waste management issues. Our program will support the expansion of bioenergy production facilities, particularly in rural and economically disadvantaged regions. This initiative will create jobs, stimulate local economies, and provide a renewable energy source that is both sustainable and scalable. We will ensure that bioenergy production adheres to rigorous sustainability criteria, minimizing environmental impact and avoiding competition with food production.

High transport electrification: The transportation sector is a major contributor to carbon emissions in Europe. Our program prioritizes high transport electrification to combat this issue. We will accelerate the adoption of electric vehicles (EVs) by expanding charging infrastructure, providing incentives for EV purchases, and investing in the electrification of public transportation. Furthermore, we will support the development of next-generation batteries and explore sustainable mobility solutions, such as hydrogen fuel cells for heavy-duty transport. By focusing on the electrification of both personal and public transport, we will significantly reduce emissions, improve air quality, and enhance the quality of life for European citizens.

Middle to High heat electrification: Heating is a significant energy demand in Europe, and transitioning to electrified heating systems is essential for reducing carbon emissions. Our program advocates for middle to high heat electrification, particularly in industrial processes and residential heating. We will pro-

mote the adoption of advanced heat pump technologies, electric boilers, and other efficient electric heating solutions. By doing so, we will decrease our reliance on fossil fuels, increase energy efficiency, and support the development of a renewable-powered grid. Research and innovation in heat electrification technologies will be a key focus, ensuring these solutions are both effective and economically viable.

Middle to Low imports: To achieve energy self-sufficiency and security, our program emphasizes middle to low imports of energy. By maximizing domestic renewable energy production and optimizing storage capacity, we can reduce our dependence on external energy sources. However, strategic partnerships and interconnections with neighboring countries will remain essential to balance supply and demand and ensure energy stability. This approach will enhance our resilience to energy market fluctuations and geopolitical risks, fostering a more stable and self-reliant Europe.

Conclusion: Our political program for a Green Transition represents a visionary and comprehensive approach to building a sustainable future for Europe. By focusing on high storage capacity, significant bioenergy utilization, extensive transport electrification, and strategic heat electrification, while maintaining moderate imports, we will lead Europe into a new era of environmental and economic prosperity. This strategy addresses the immediate challenges of the climate crisis and lays the groundwork for long-term sustainability and resilience. Together, we can build a greener, healthier, and more equitable Europe for present and future generations.

5 Supplemental Tables

Table S1: Ranges of variation under the variant 2C_SSP2, considered in the main paper, compared to the ranges in all the 3 variants in [3], with respect to the outputs of interest. Changes are marked in bold.

	2C_SSP2		all 3 variants	
output of interest	min	max	min	max
Renewables	278 EJ/yr.	1119 EJ/yr.	**274 EJ/yr.**	**1245 EJ/yr.**
Fossils	238 EJ/yr.	1279 EJ/yr.	**205 EJ/yr.**	1279 EJ/yr.
Electric industry	16%	71%	16%	**72%**
Electric heating	14%	89%	14%	89%
Non-fossil transport	46%	87%	46%	**91%**

6 Supplemental Figures

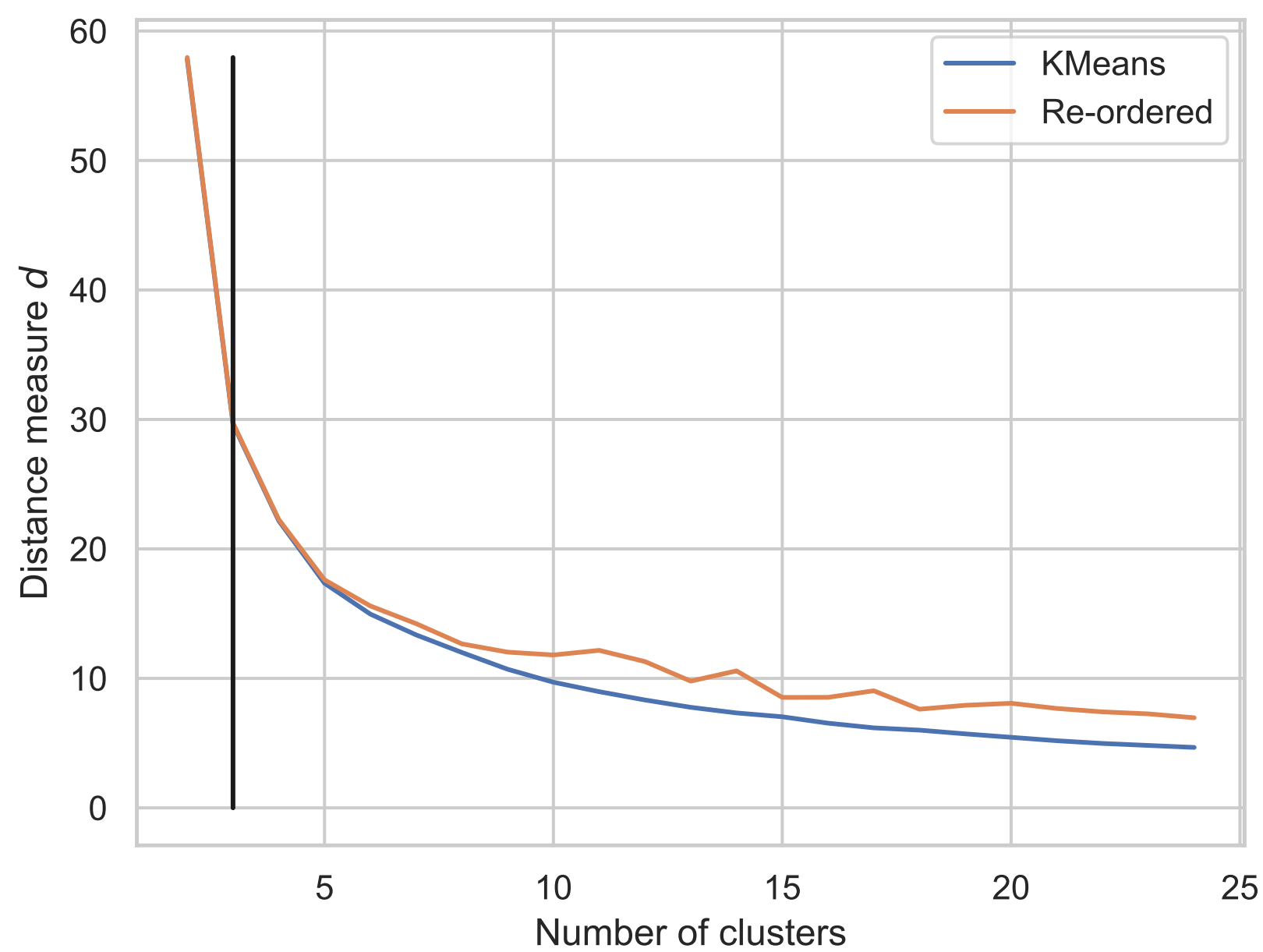

Figure S1: Elbow curve for the global case study under uncertainty showing the distance measure d as defined in Equation (3) of the main paper. The elbow curve is shown for both the initial k-Means clustering as well as for the re-ordered clusters (see Figure 6 in the main paper). For $k = 3$, the reordering of points results in an increase in the distance measure d from 29.64 to 29.73 (0.3% increase).

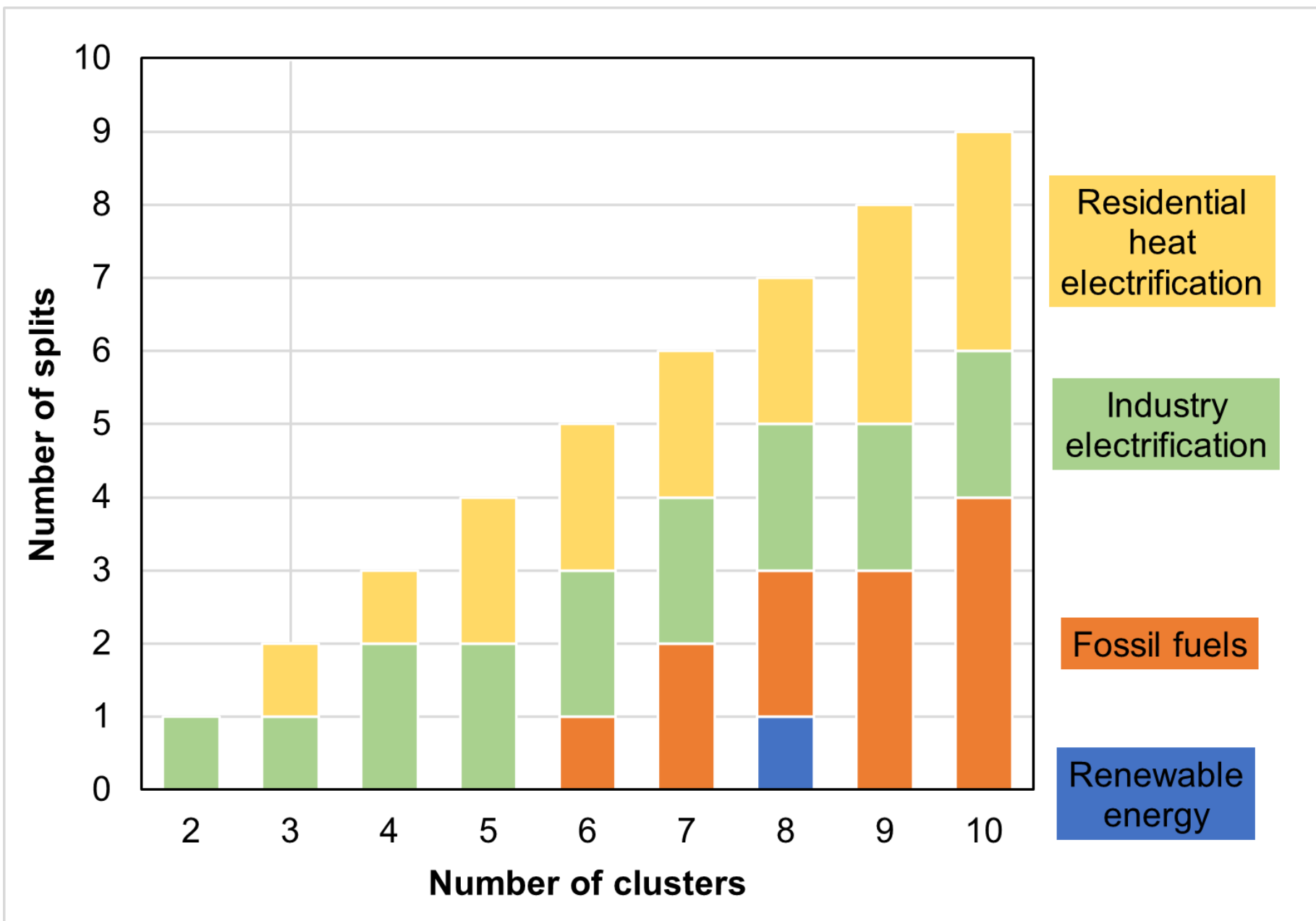

Figure S2: Number of splits depending on the number of clusters k as performed by the classification tree on the outputs of interest. For up to ten clusters, the tree never splits on the share of non-fossil transport.

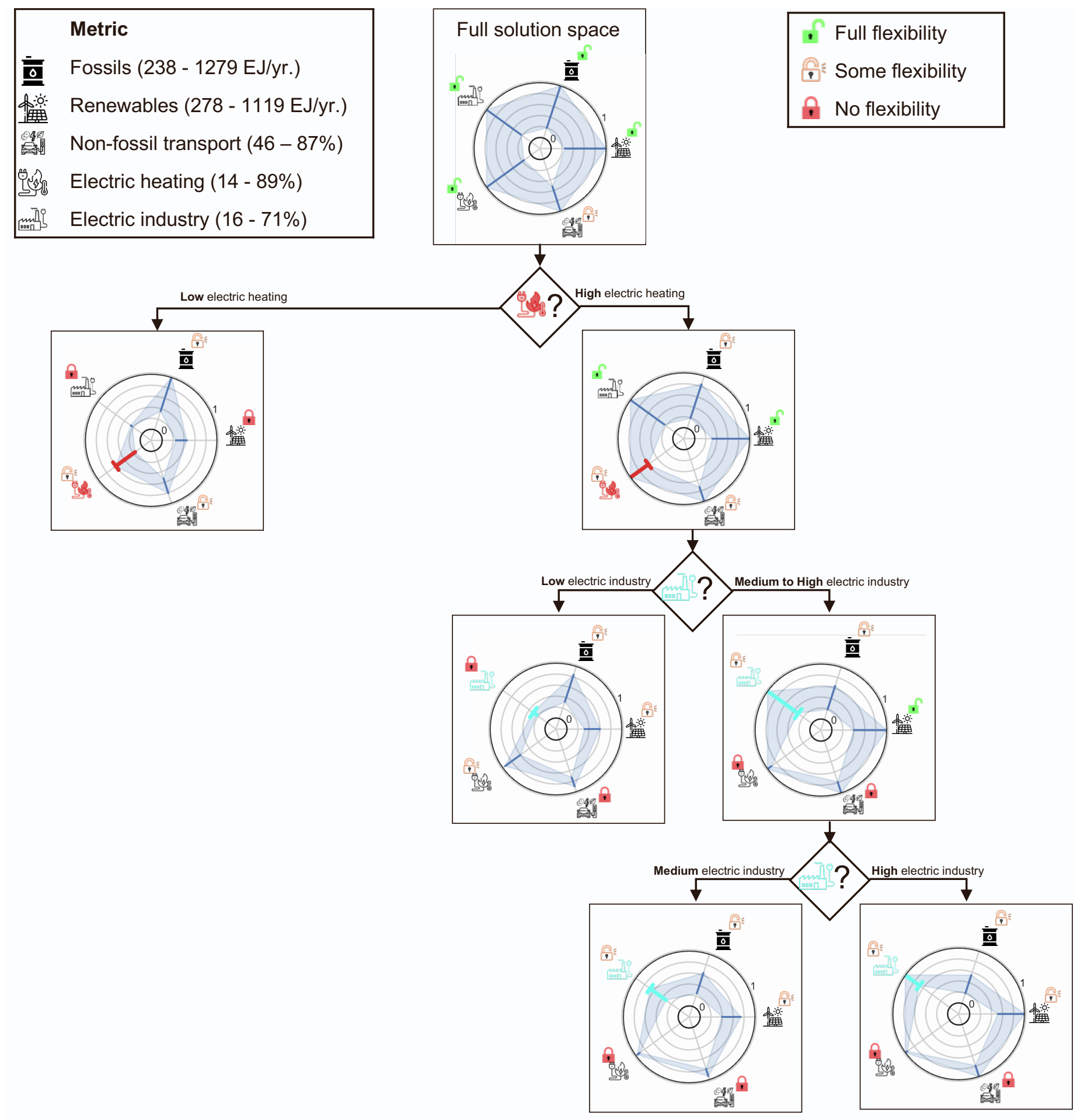

Figure S3: Decision tree for the global case study with a fourth cluster (compare to Figure 3 in the main paper).

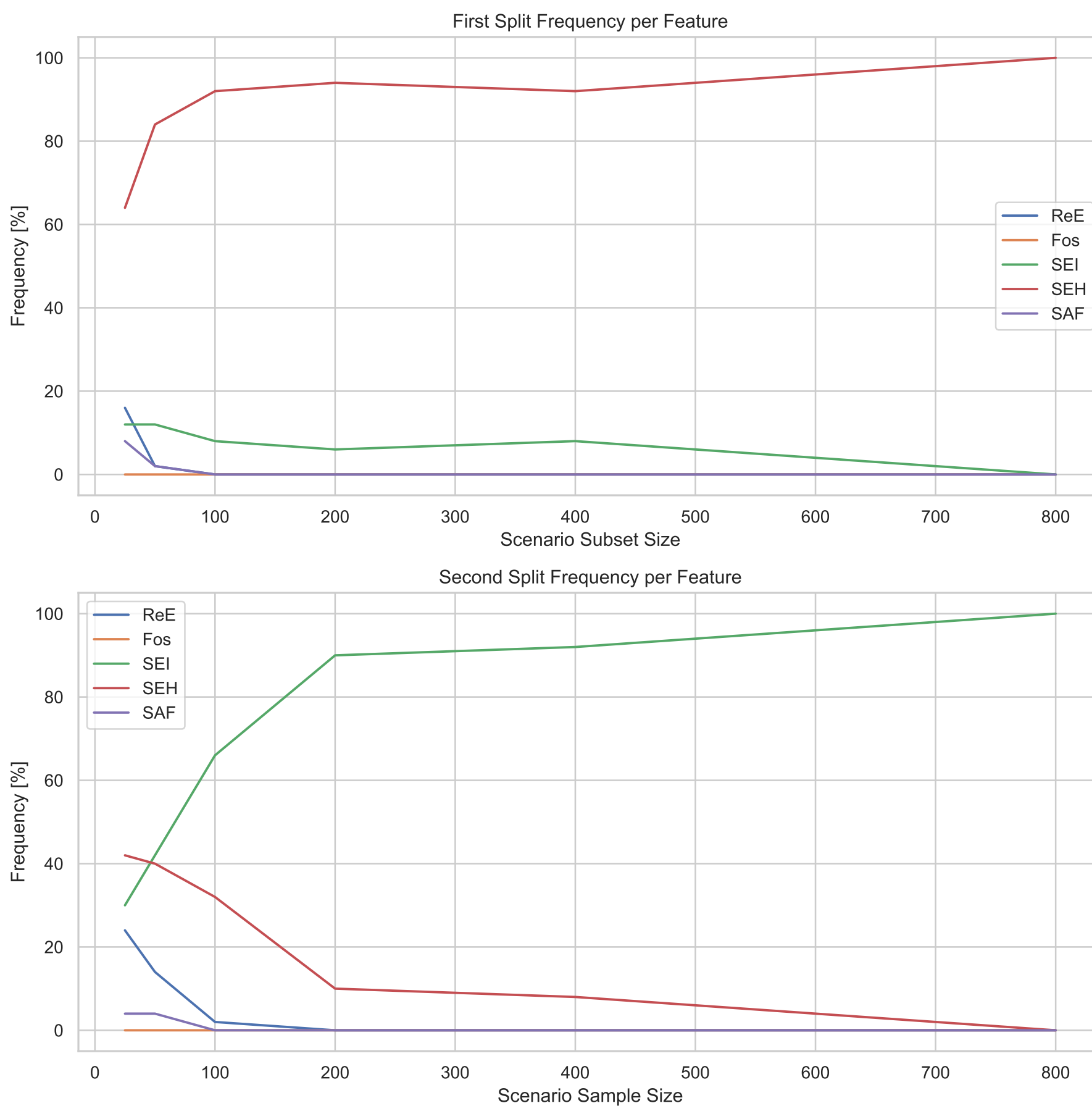

Figure S4: First (TOP) and second (BOTTOM) splits of the decision trees for the global case study under uncertainty (Figure 3 in the main paper) with increasing size of scenario subsets used to train the tree (25; 50; 100; 200; 400; 800). For each scenario sample size, the sampling is repeated 50 times to account for the stochasticity of the sampling process (ReE: Renewables; Fos: Fossils; SEI: Electric Industry; SEH: Electric Heating; SAF: Non-Fossil Transport).

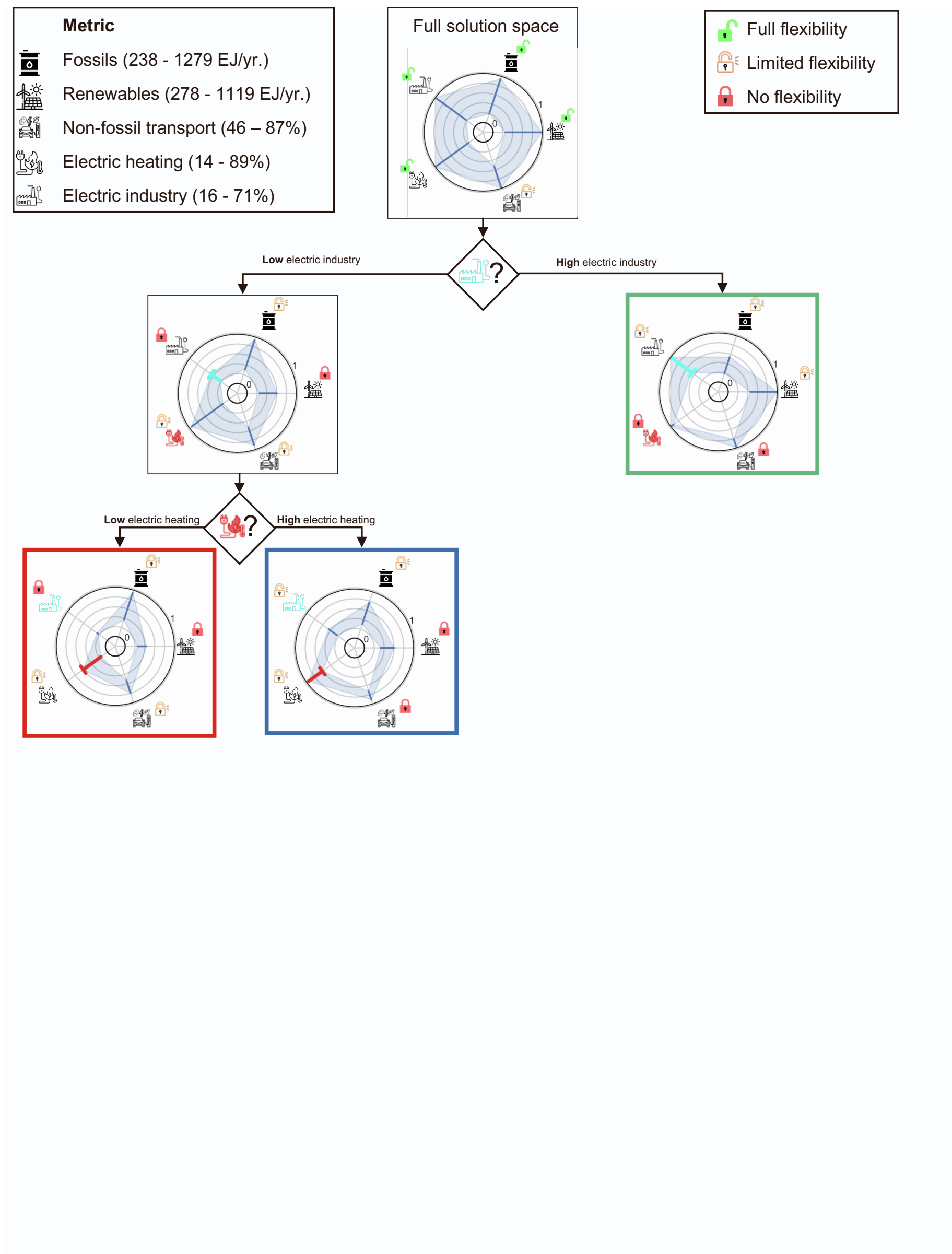

Figure S5: Decision Tree for the global case study under uncertainty (scenario sample size = 400), representing an instance in which the tree first splits on “Electric Industry” and second on “Electric Heating”.

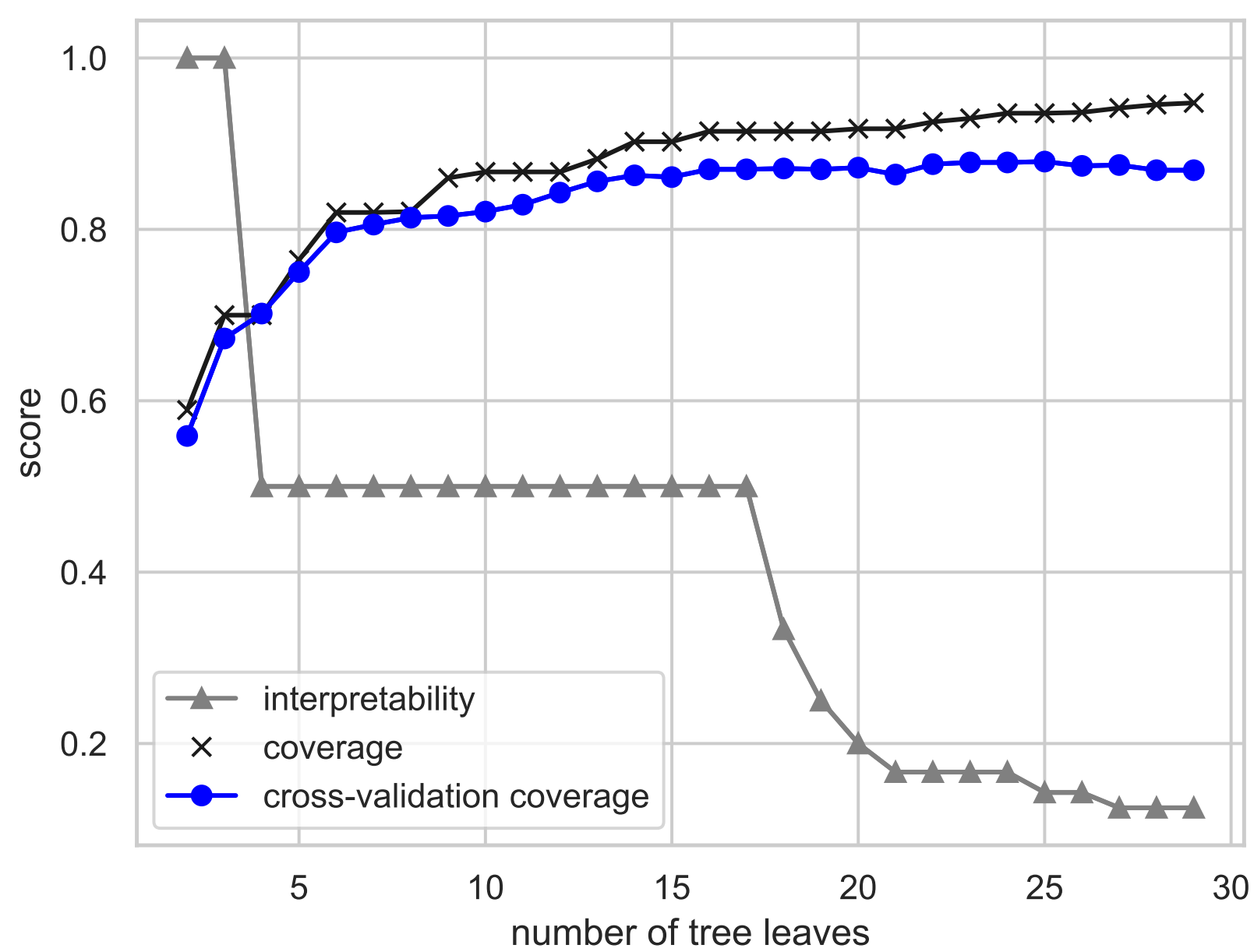

Figure S6: Interpretability score, defined as one over the number of variables that the classification tree branches on, vs. coverage score, defined as the fraction of points that are correctly assigned to their clusters by the classification tree. Moreover, the coverage of a 5-fold cross-validation analysis is shown.

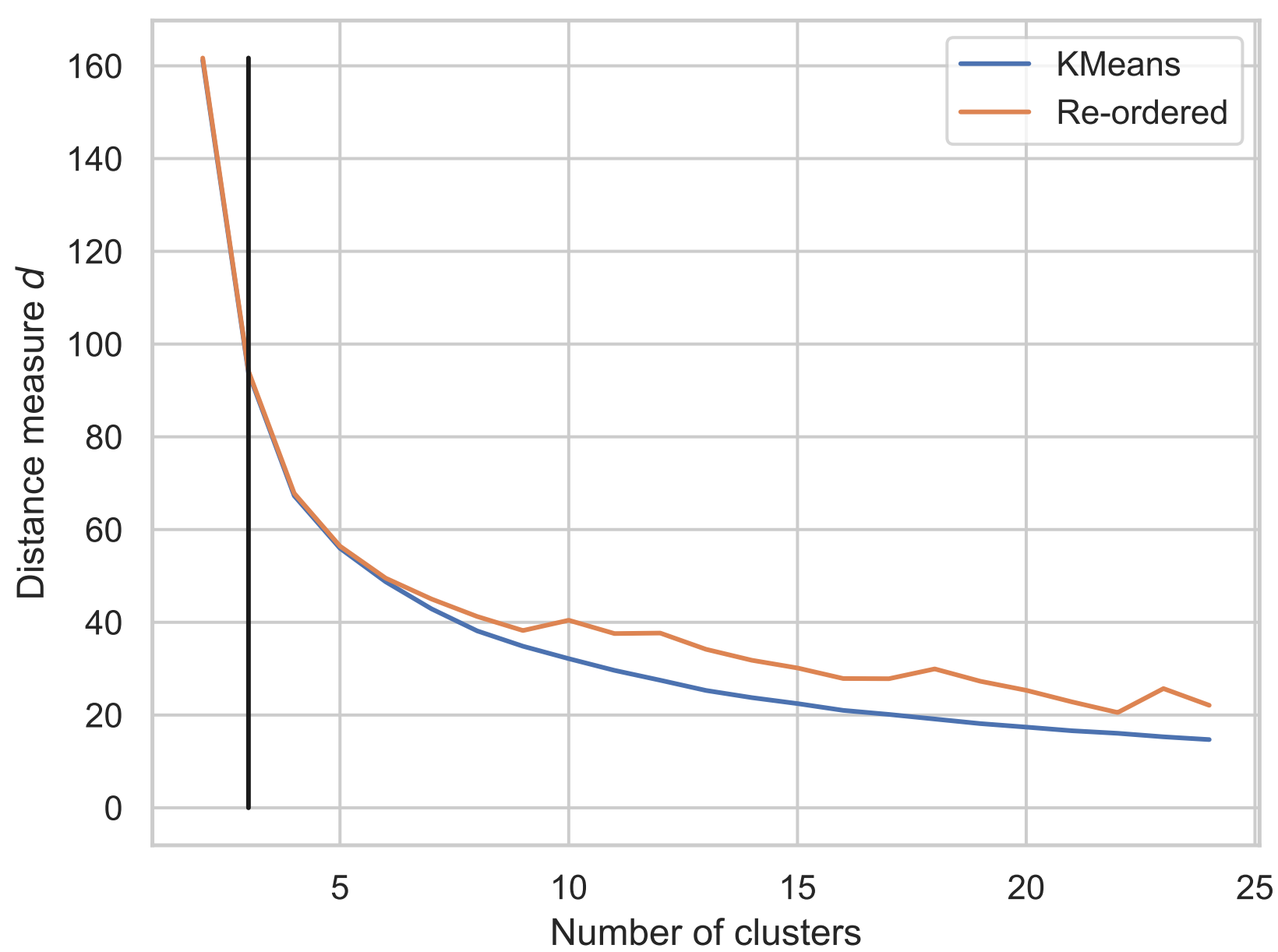

Figure S7: Elbow curve for the global case study with all three variants included showing the distance measure d as defined in Equation (3) of the main paper. The elbow curve is shown for both the initial k-Means clustering as well as for the re-ordered clusters (see Figure 6 in the main paper).

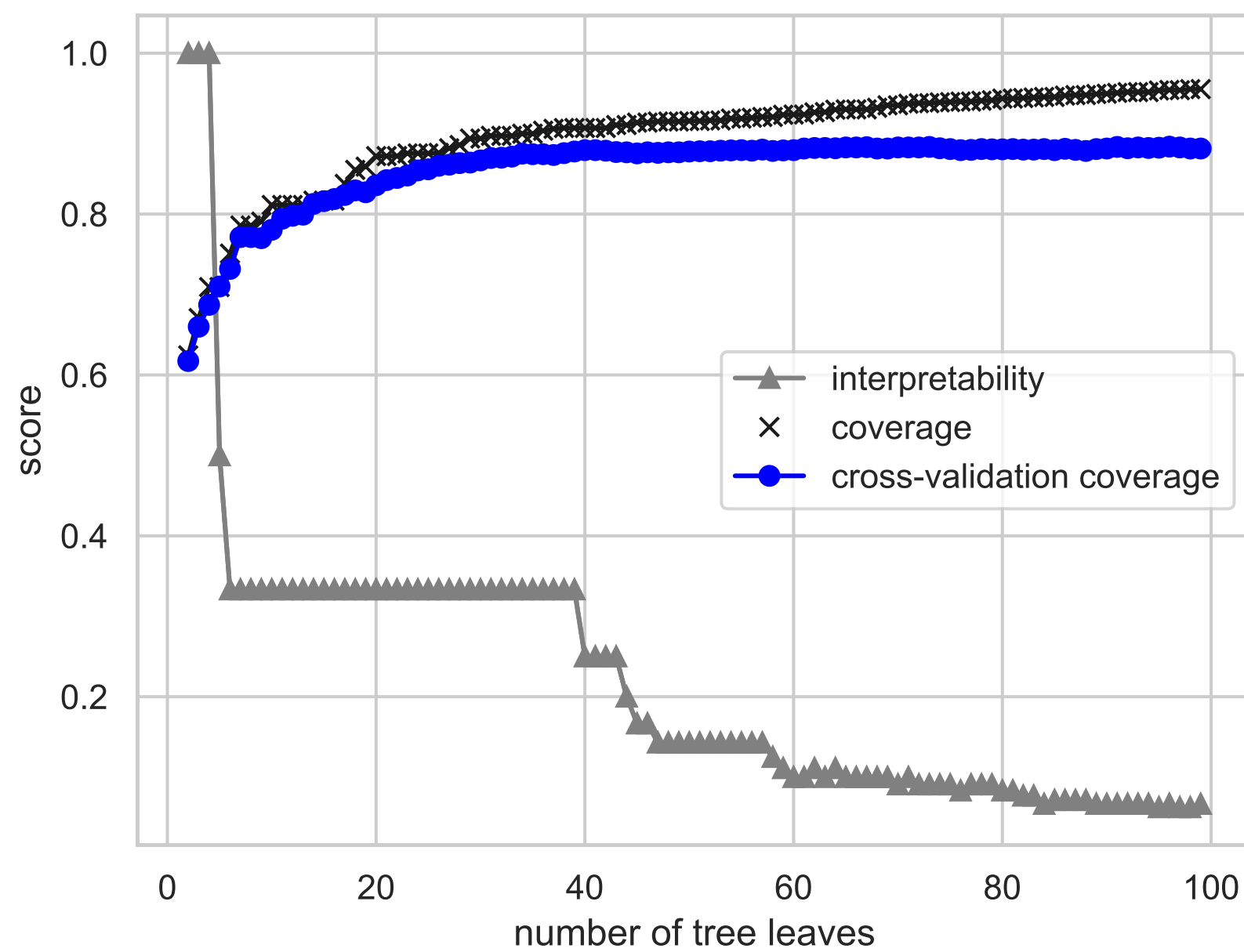

Figure S8: Scenario discovery analysis for the global case study under uncertainty using all three variants: Interpretability score, defined as one over the number of variables that the classification tree branches on, vs. coverage score, defined as the fraction of points that are correctly assigned to their clusters by the classification tree. Moreover, the coverage of a 5-fold cross-validation analysis is shown.

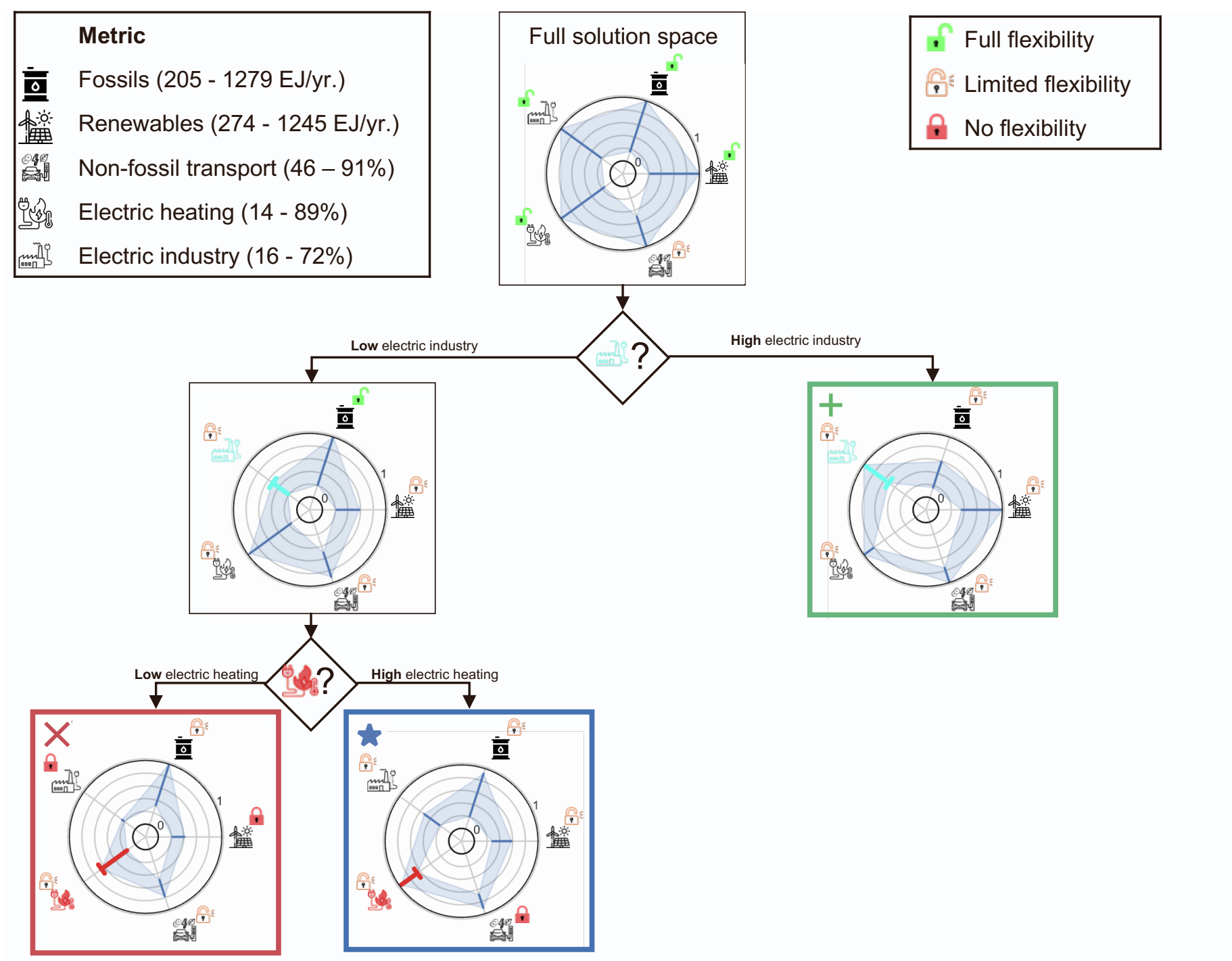

(a)

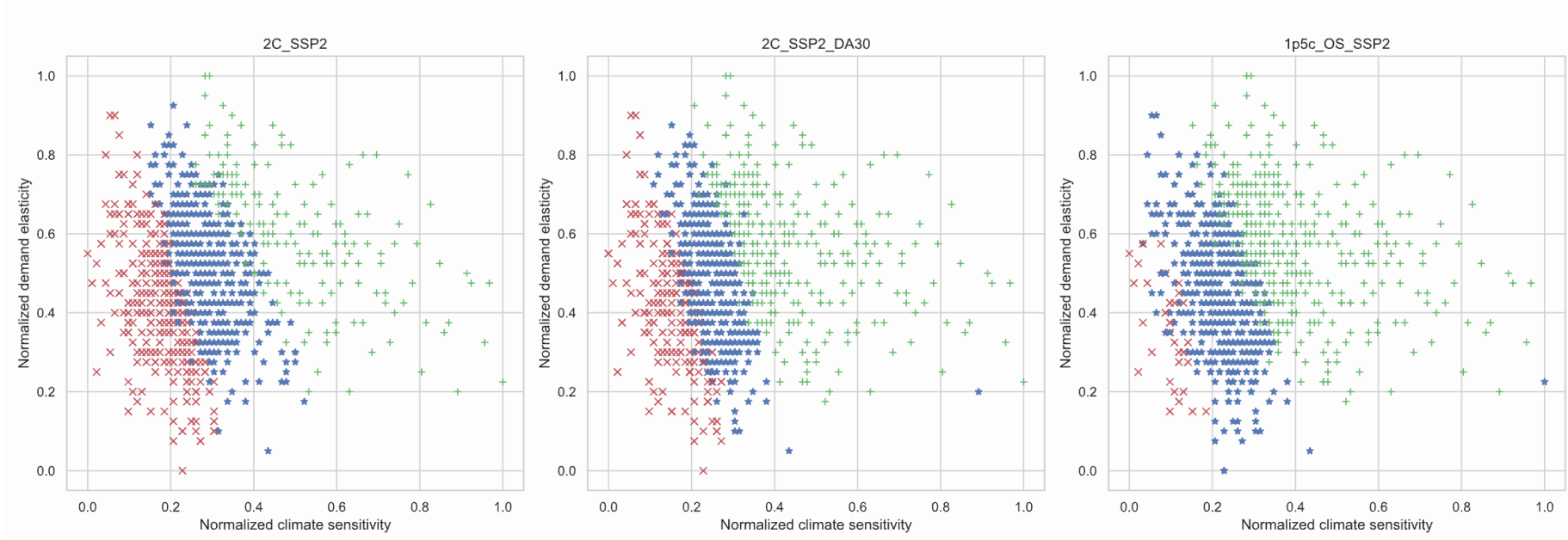

(b)

Figure S9: (a) Decision tree translating 3000 scenarios for the global energy transition in 2100 into three storylines determined by two key drivers (electrification of heating and industry) along five outputs of interest in analogy to Figure 3 in the main paper. (b) Clustering of the 3000 scenarios along the two main uncertain drivers and the variant. The coloring links the scatter plot to the three storylines of the tree in (a): Scenarios with high penetration of fossils and low sector coupling assume low climate sensitivity and low demand elasticity.

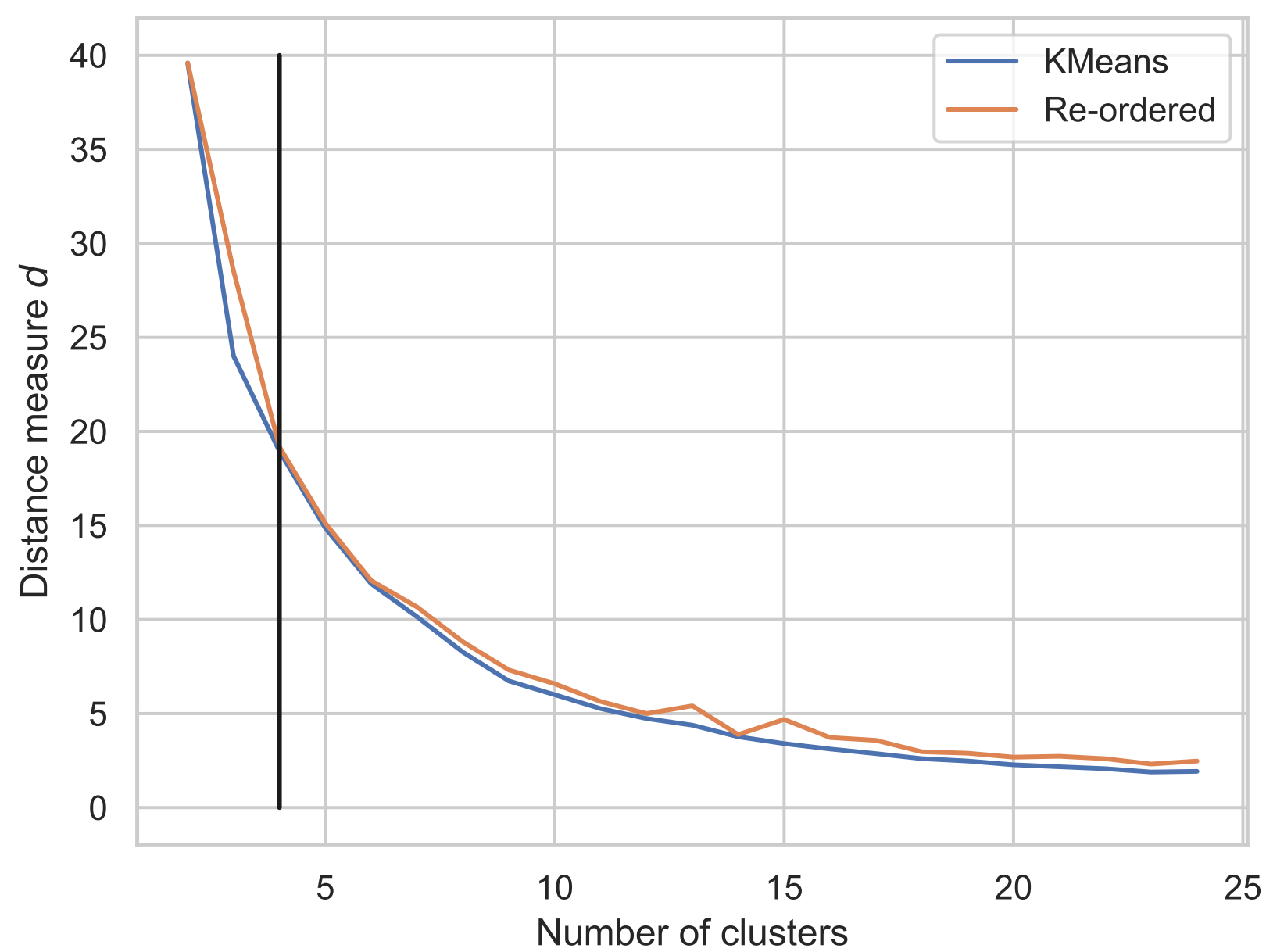

Figure S10: Elbow curve for the European case study showing the distance measure d as defined in Equation (3) of the main paper. The elbow curve is shown for both the initial k-Means clustering as well as for the re-ordered clusters (see Figure 6 in the main paper). For $k = 4$, the reordering of points results in a decrease in accuracy from 19.0 to 19.2.

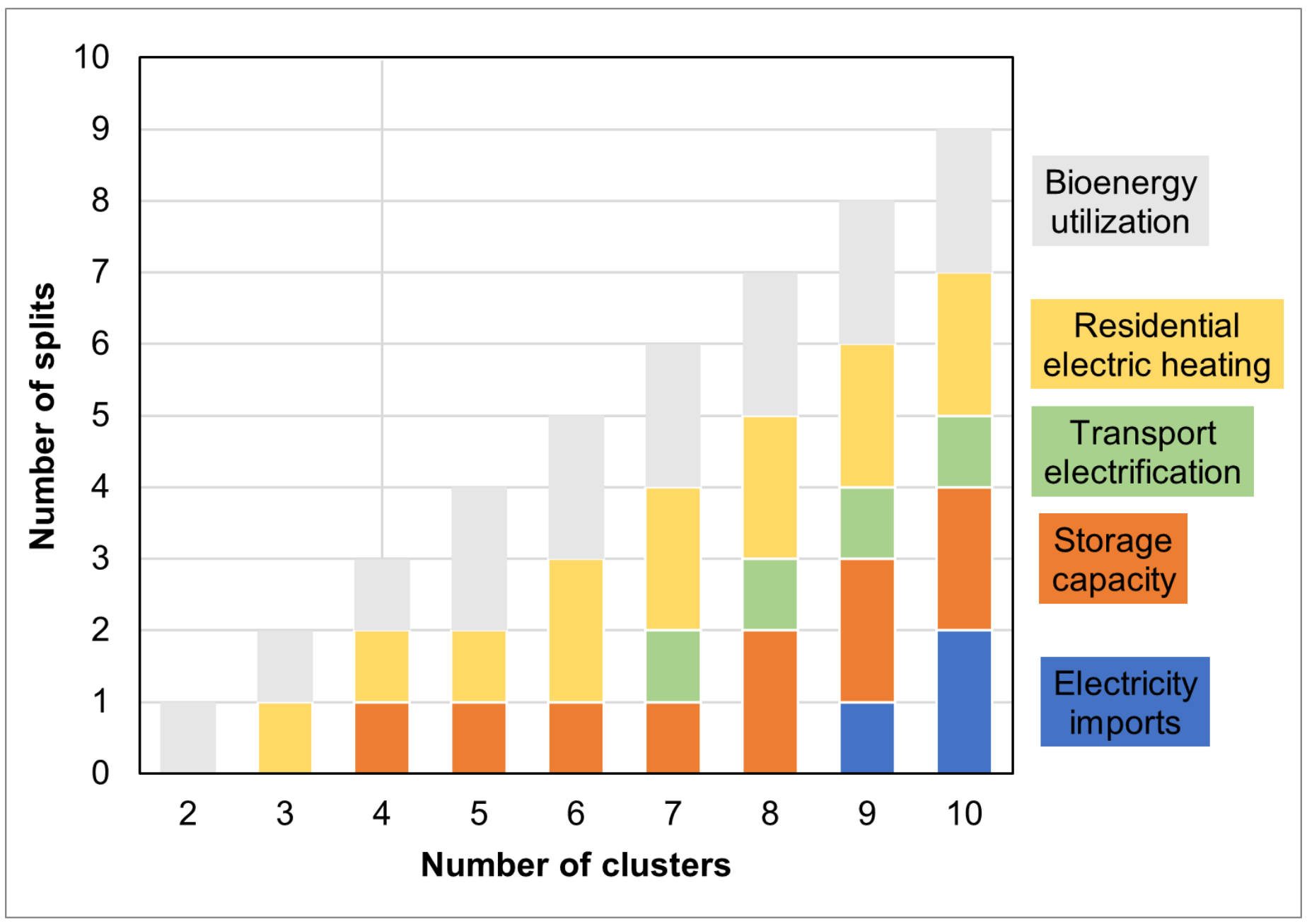

Figure S11: Number of splits depending on the number of clusters k as performed by the classification tree on the outputs of interest.

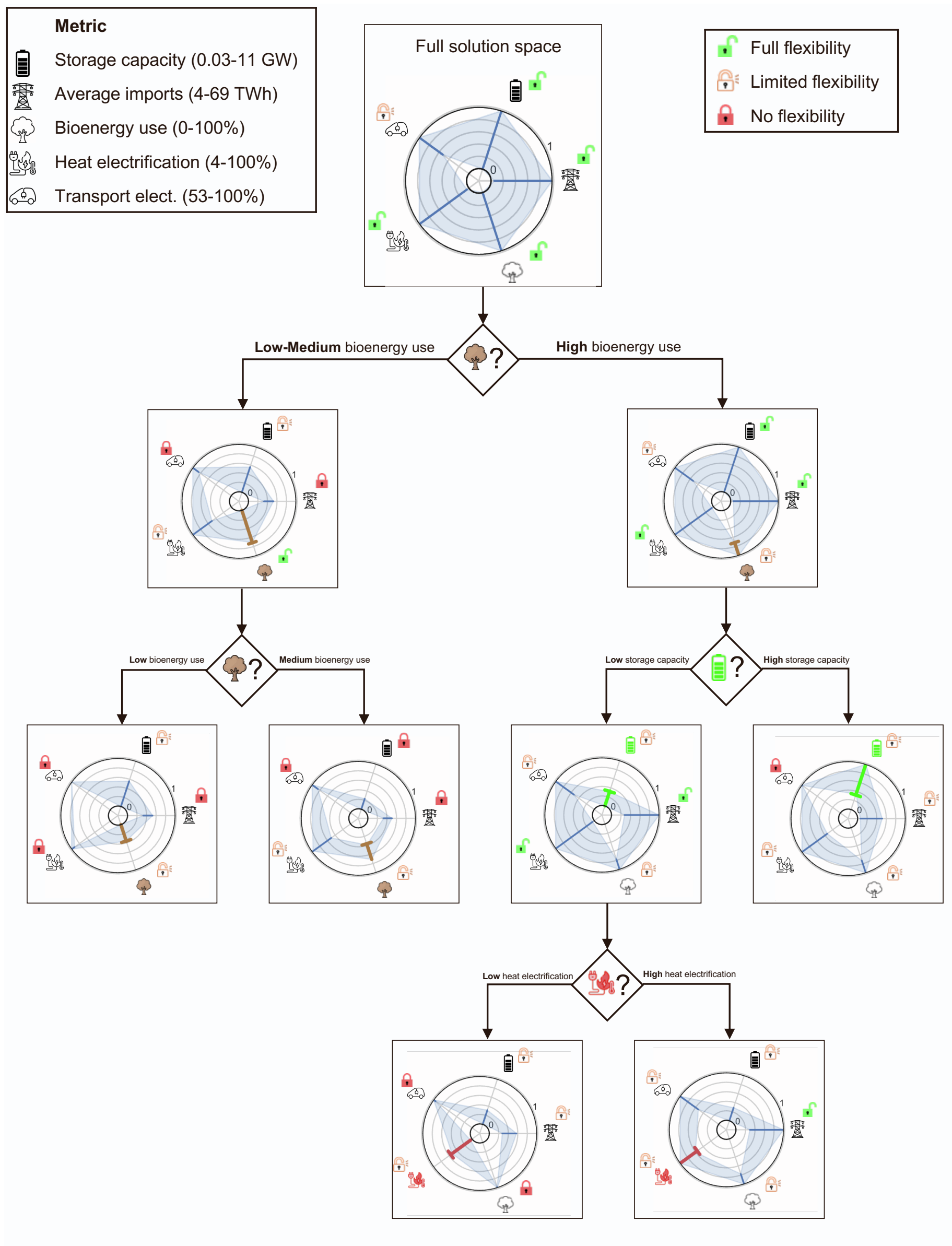

Figure S12: Decision tree for the European case study with a fifth cluster (compare to Figure 4 in the main paper).

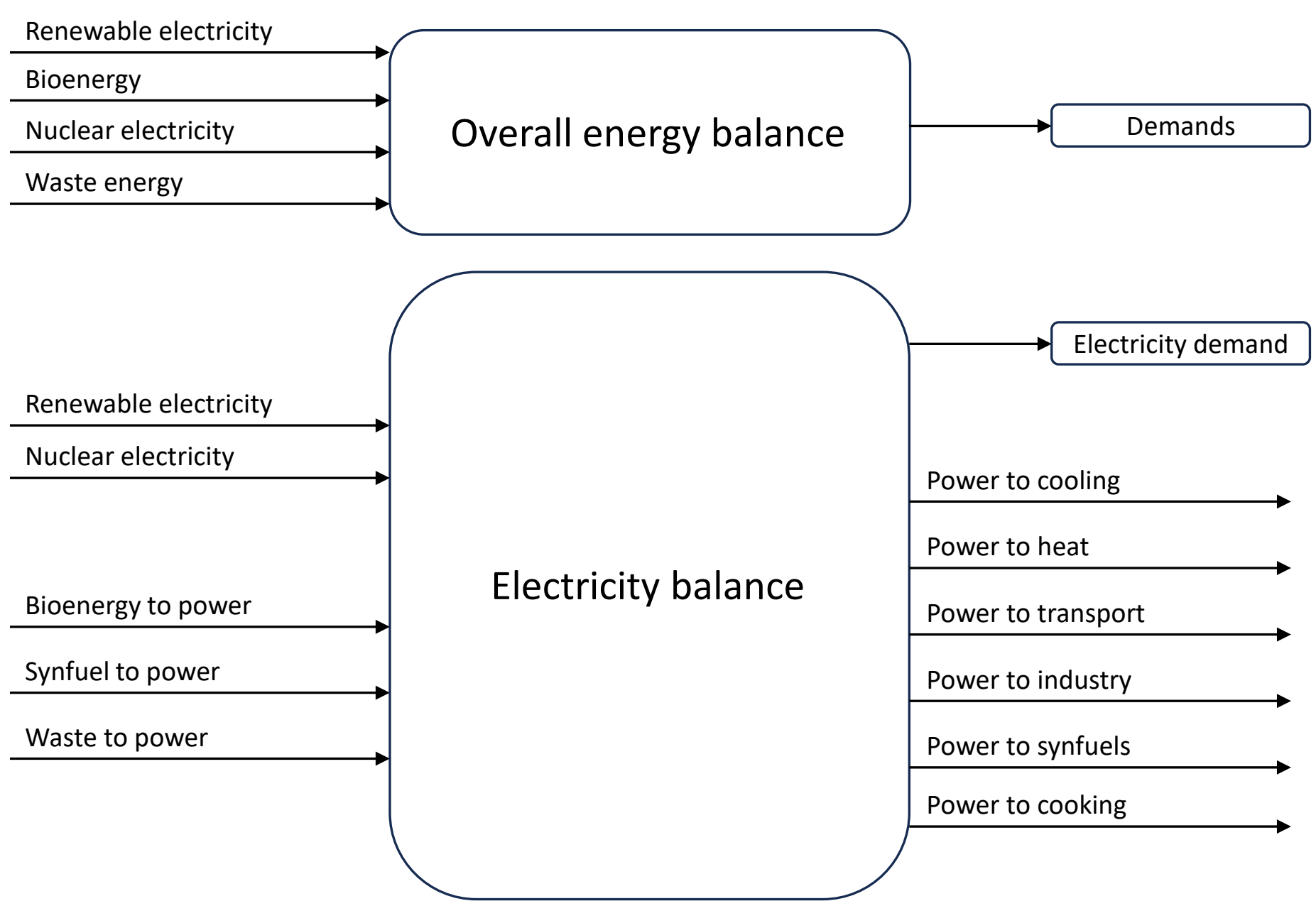

Figure S13: Considered flows in the overall energy and electricity balance for systematically choosing outputs of interest in the European case study.

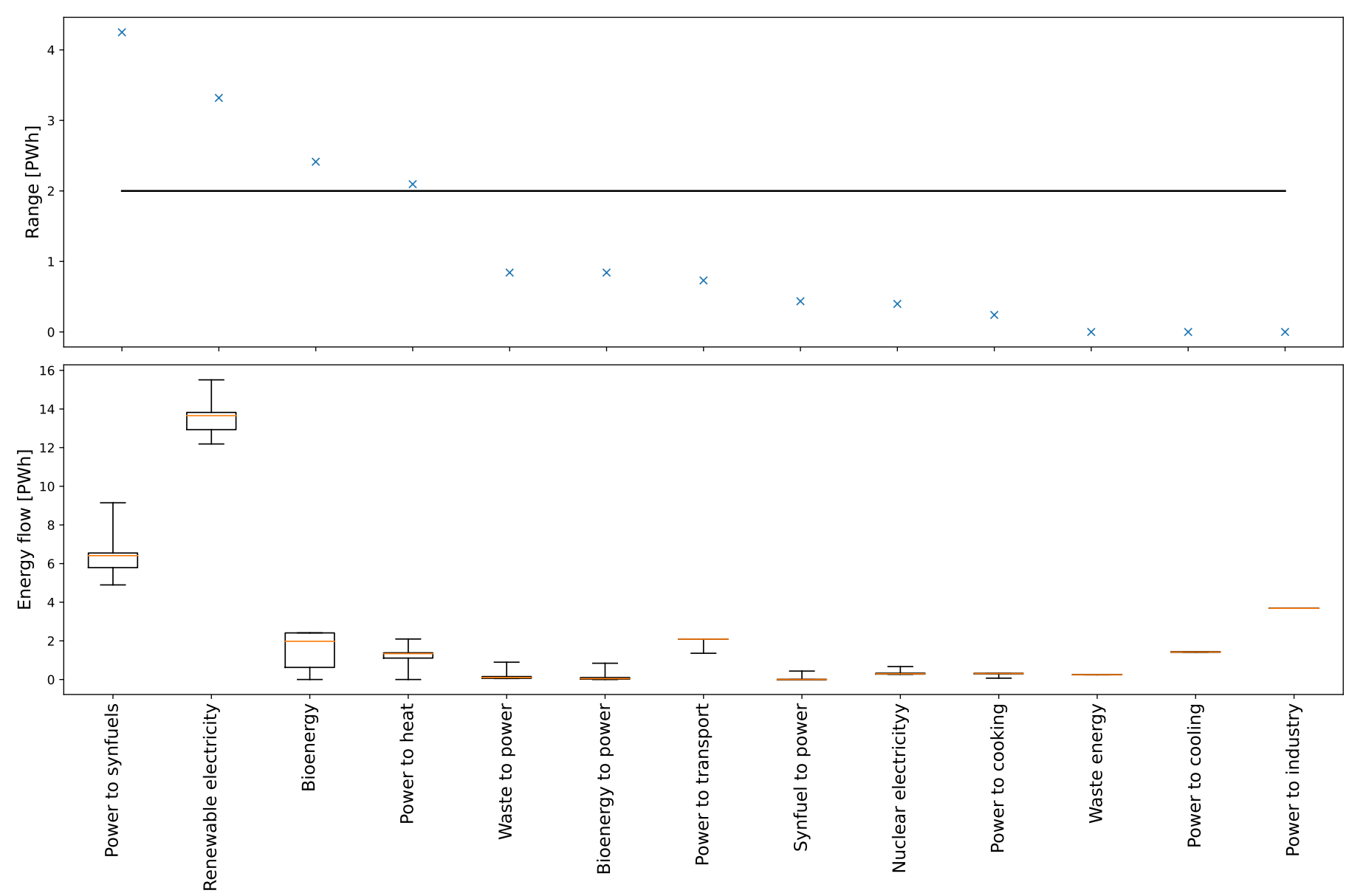

Figure S14: Ranges of variation (top) and boxplots (bottom) of the flows in the overall energy and electricity balance over all 441 scenarios in the European case study. The four flows with a range above 2 PWh are chosen as outputs of interest (see the black line in the upper plot).

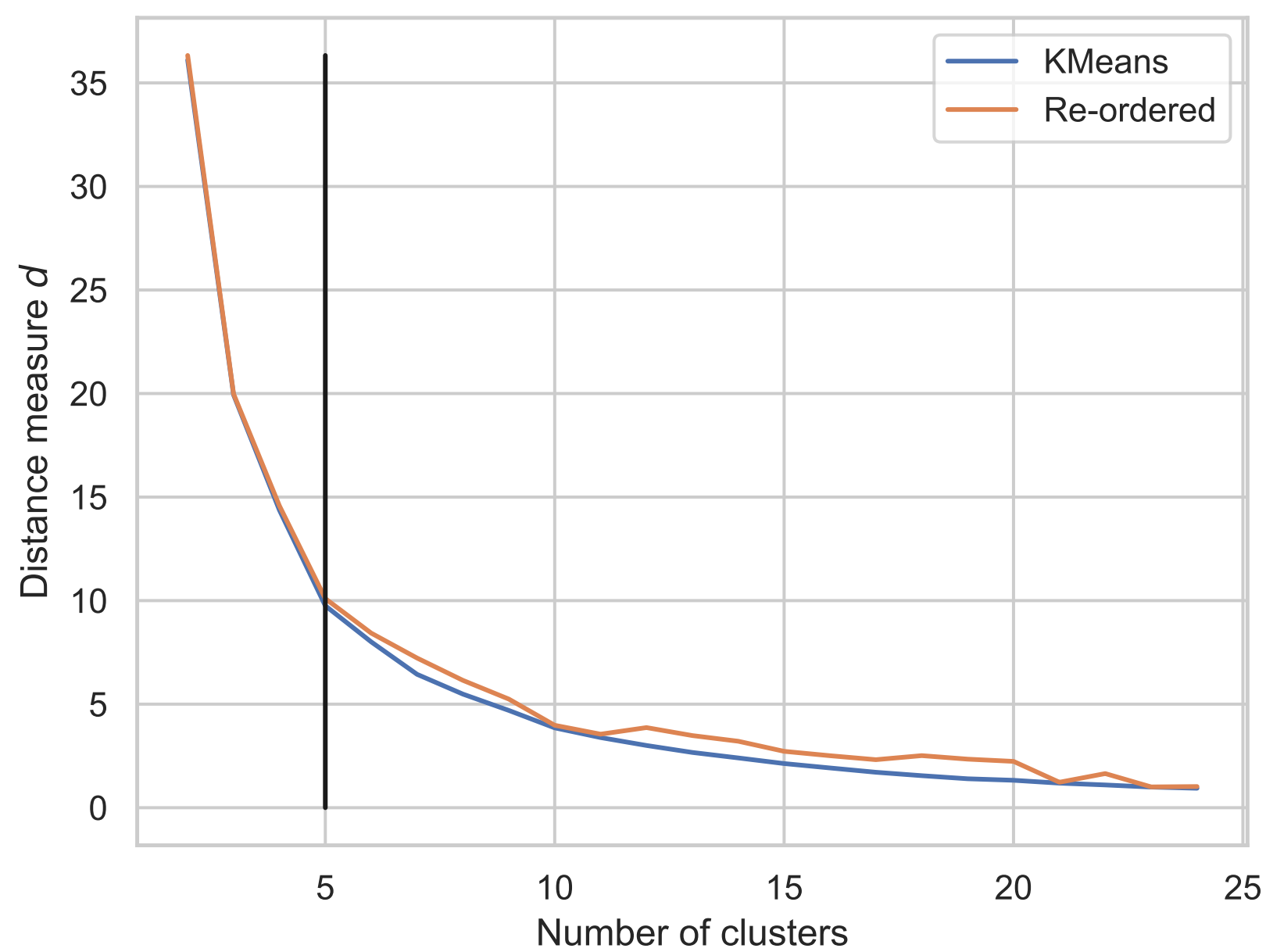

Figure S15: Elbow curve for the European case study with outputs of interest chosen from balances showing the distance measure d as defined in Equation (3) of the main paper. The elbow curve is shown for both the initial k-Means clustering as well as for the re-ordered clusters (see Figure 6 in the main paper).

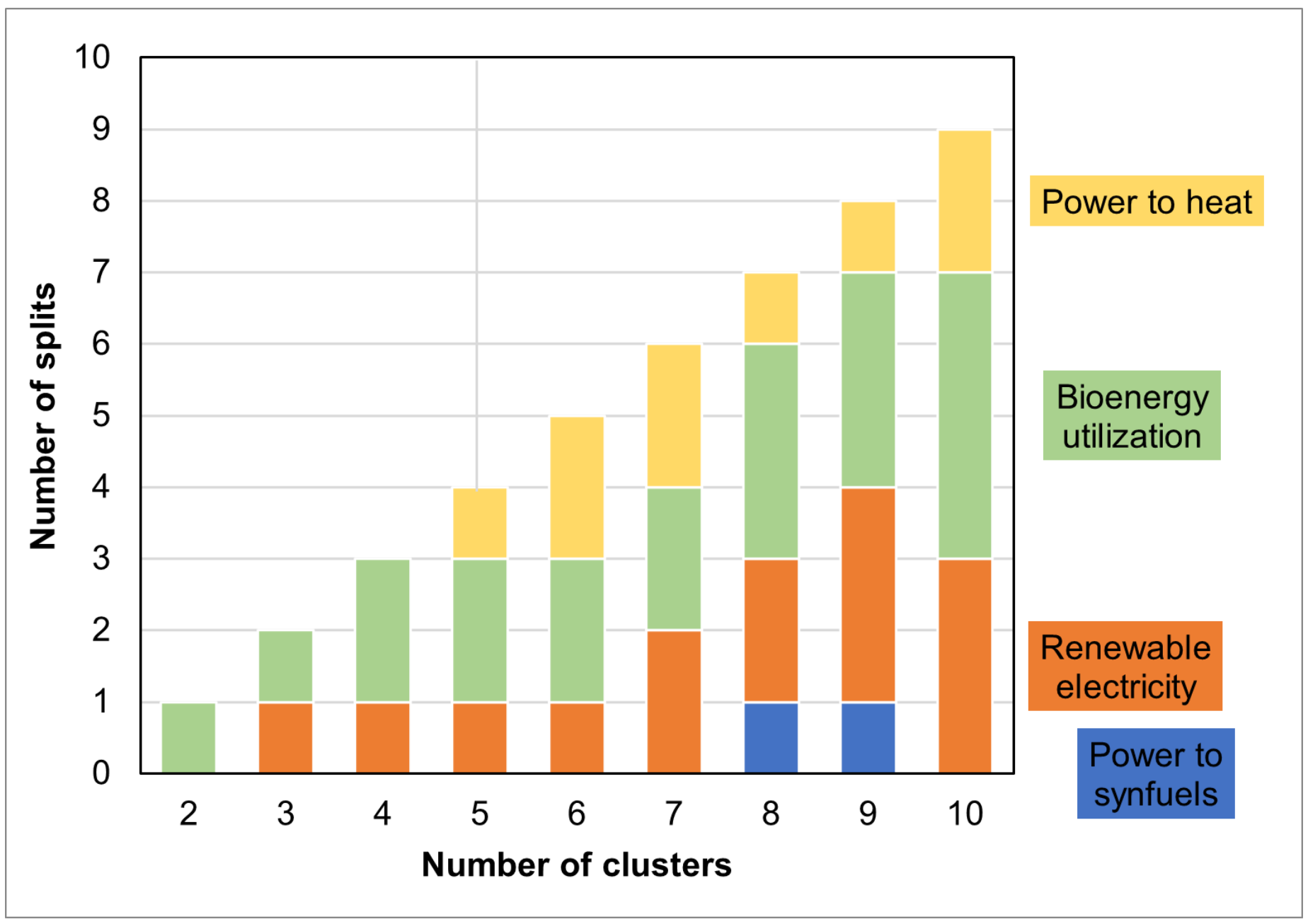

Figure S16: Number of splits depending on the number of clusters k as performed by the classification tree on the outputs of interest.

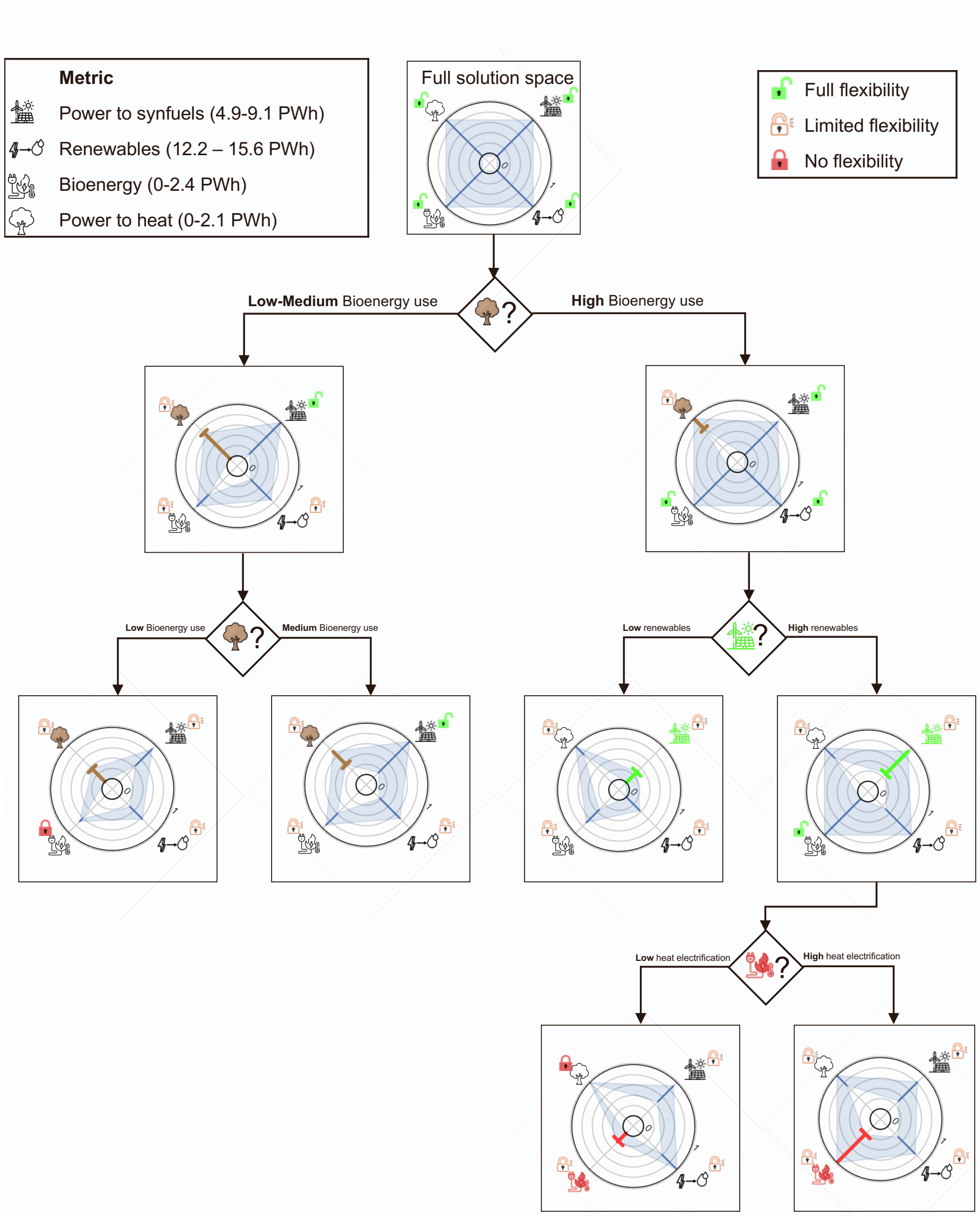

Figure S17: Decision tree for the European case study with outputs of interest chosen based on energy balances.

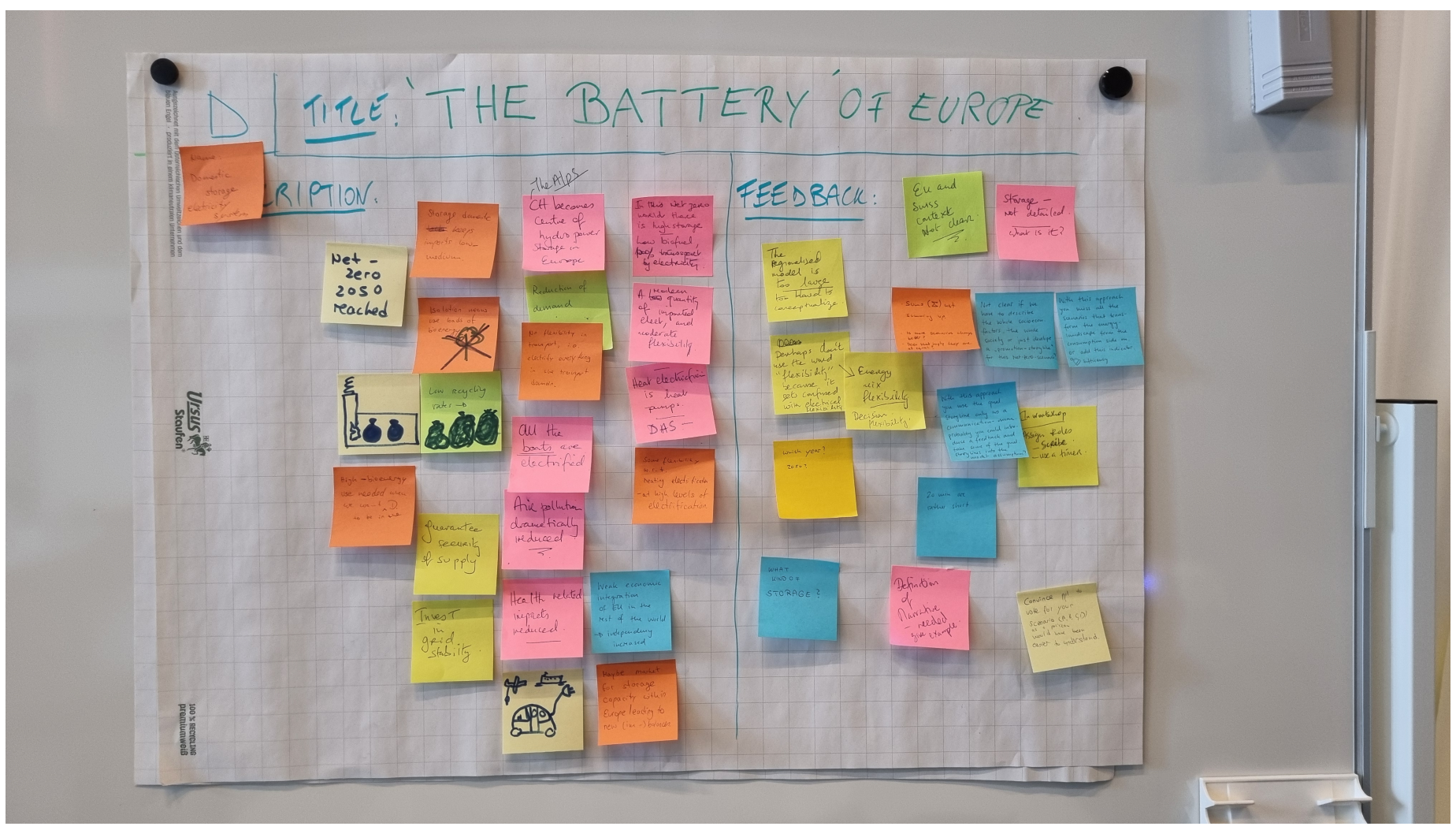

Figure S18: SSH Workshop, Zurich, June 17-18. Example output by the group focusing on Storyline D, defined by high use of bioenergy and high deployment of storage in Figure 4 (main paper).

References

[1] Groissböck, M. Are open source energy system optimization tools mature enough for serious use? *Renewable and Sustainable Energy Reviews* **102**, 234–248 (2019).

[2] Open Energy Modelling Initiative. Open Energy Modelling Initiative. https://openmod-initiative.org (2018).

[3] Panos, E. *et al.* Deep decarbonisation pathways of the energy system in times of unprecedented uncertainty in the energy sector. *Energy Policy* **180**, 113642 (2023).

[4] Gerst, M. D., Wang, P. & Borsuk, M. E. Discovering plausible energy and economic futures under global change using multidimensional scenario discovery. *Environmental Modelling & Software* **44**, 76–86 (2013).

[5] Pickering, B., Lombardi, F. & Pfenninger, S. Diversity of options to eliminate fossil fuels and reach carbon neutrality across the entire European energy system. *Joule* **6**, 1253–1276 (2022).
